# Ex-situ generation of gaseous nitriles in two-chamber glassware for facile haloacetimidate synthesis

**DOI:** 10.3762/bjoc.21.188

**Published:** 2025-11-07

**Authors:** Nikolai B Akselvoll, Jonas T Larsen, Christian M Pedersen

**Affiliations:** 1 Department of Chemistry, University of Copenhagen. Universitetsparken 5, DK-2100 Copenhagen O, Denmark; 2 Current Address: Technical University of Denmark, Department of Chemistry, Kemitorvet 207, DK-2800 Kgs. Lyngby, Denmark

**Keywords:** gaseous reagents, glycosyl donor, haloacetimidates, haloacetonitrile, two-chamber reactor

## Abstract

The synthesis of fluorinated haloacetimidates relies on the access to the corresponding fluoroacetonitriles, which are toxic gaseous molecules difficult to store and handle. In this work we develop a safe two-chamber method for the ex-situ generation of these reagents in one chamber and their subsequent reaction with *O*-nucleophiles in the second chamber. The method is easy to setup, control and gives access to new haloacetimidates under mild conditions, similar to the ones used for the synthesis of the more commonly used trichloroacetimidates.

## Introduction

Trifluoroacetonitrile is an electrophilic reagent that has seen a variety of uses, mostly for incorporating trifluoromethyl groups into organic compounds [1]. As an example it has been successfully utilized for the synthesis of various trifluoromethyl-containing aza-heterocycles, such as pyridines [2], pyridinones and pyrimidinones [3], tetrazoles [4], tetrazapentalenes [5] and uracil [6]. In addition, it has also been used for the synthesis of fluoro-substituted ketones [7] and substituted trifluoroacetamides via the acetimidates through the Overman rearrangement [8–9].

Trifluoroacetonitrile is a highly toxic gas being of limited commercial availability and it can furthermore be difficult to get from the few commercial suppliers due to transport restrictions. This, together with local restrictions, because of the inherent danger of working with this toxic gas, restricts its use in laboratory scale synthesis.

**Figure 1:**
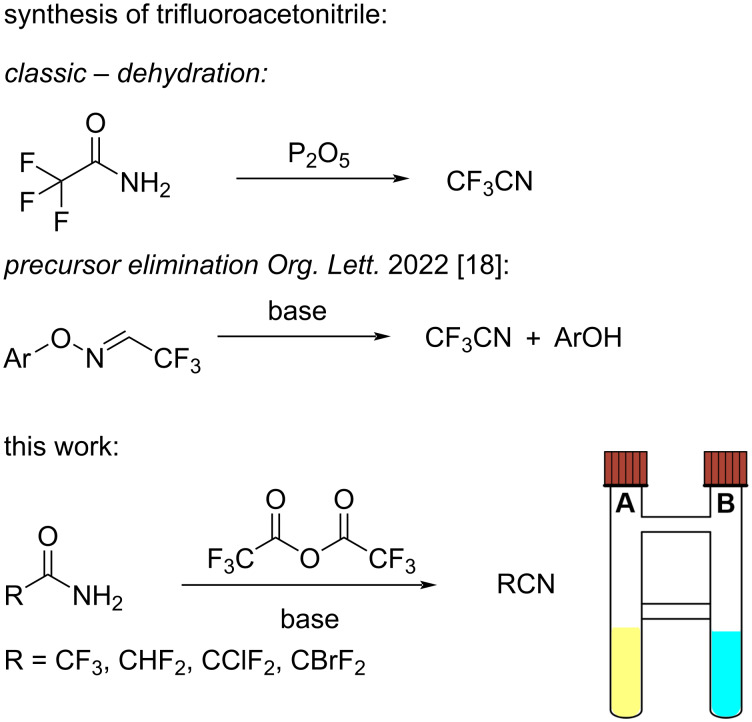
Examples of methods for the synthesis of trifluoroacetonitrile and our set-up using a two-chamber reactor.

Consequently, when used in organic synthesis, trifluoroacetonitrile is typically synthesized on demand. The most common method is by dehydration of trifluoroacetamide using Gilman’s method with phosphorous pentoxide [10] (Figure 1), Swern conditions [11–12], PPh_3_ and CCl_4_ under basic conditions or by using triﬂuoroacetic anhydride in pyridine [13]. Other methods involving HF or small fluorinated gaseous compounds at high temperatures have been developed, but are not practical in a standard research laboratory [14–17]. Recently, 2,2,2-trifluoroacetaldehyde *O*-(aryl)oxime has been introduced as a precursor for trifluoroacetonitrile allowing to work under mild conditions at room temperature (Figure 1) [18]. Whether one prepares a precursor or the actual reagent, a separate setup is needed and hence handling, storage, and transfer of this toxic gas becomes a safety issue.

To enable the facile and safe use of trifluoroacetonitrile, and other partially fluorinated acetonitriles, we set out to develop a methodology, which enabled facile use of these gases based on Parker’s method [13], i.e., generating trifluoroacetonitrile from trifluoroacetamide through dehydration with trifluoroacetic anhydride. The usual procedure would be to synthesize the gas in a different reaction vessel and use it directly via transfer as a gas or in solution. Alternatively, it can be stored in a cold trap (trifluoroacetonitrile boiling point = −64 °C). To minimize the setup needed and handling we decided to utilize the two-chamber reaction vessels introduced by Skrydstrup and co-workers [19], which were originally designed for use with carbon monoxide reactions [20] and are now commercially available under the tradename COware (Figure 1) [21]. The two-chamber reaction vessels have found use in many cases where one of the reagents is a highly reactive, toxic, or isotopically labeled gas [22].

To test the two-chamber method for the synthesis of acetimidates, we decided to synthesize a series of haloacetimidates with varied substitution patterns. We began with carbohydrates, as the products are analogs of the trichloroacetimidates, which constitute the most commonly used class of glycosyl donors for glycoside synthesis [23]. Their trifluoro-analogs have, however, only been scarcely studied due to the difficulties in handling the trifluoroacetonitrile [24]. Schmidt published the first synthesis of glycosyl trifluoroacetimidates and concluded that their glycosyl-donor properties were similar to the trichloroacetimidates, but more difficult to prepare and purify [24]. In contrast to these observations, Nakajima et al. reported the trifluoroacetimidates to be more stable than their trichloroacetimidate counterparts [25]. Since these early examples trichloroacetimidates have become one of the most common glycosyl donors used in catalytic glycosylation, whereas the trifluoroacetimidates have received much less attention.

## Results and Discussion

**Scheme 1:**
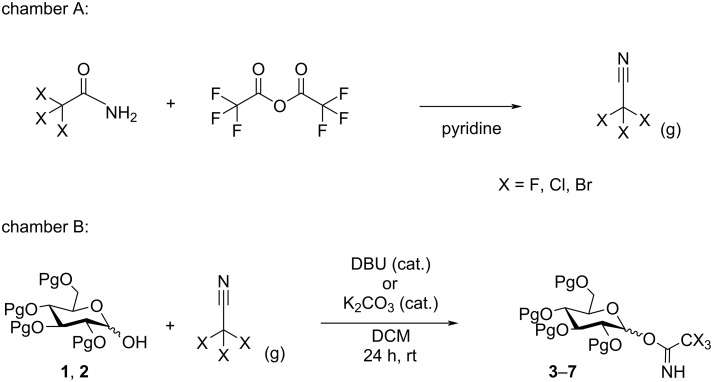
Reactions in chambers A and B. Chamber A: generation of the haloacetonitrile by dehydration of the corresponding haloacetamide. Chamber B: reaction between the generated haloacetonitrile and an *O*-nucleophile resulting in a haloacetimidate.

**Figure 2:**
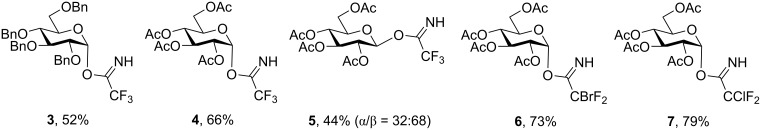
Glycosyl haloacetimidates synthesized using the two-chamber method.

We decided to synthesize some analogs of the commonly used glycosyl donors using ex-situ trifluoroacetonitrile synthesis in a two-chamber system (Figure 2 and Scheme 1). As a model system we chose 2,3,4,6-tetra-*O*-benzylglucose (**1**) and 2,3,4,6-tetra-*O*-acetylglucose (**2**), as these are among the most commonly used building blocks in glycosylations. For the synthesis chamber (A) was loaded with trifluoroacetamide (6 equiv) in pyridine. In the other chamber (B), the hemiacetal (ca. 500 mg, 1 equiv) was dissolved in CH_2_Cl_2_ together with a catalytic amount of DBU. Upon addition of trifluoroacetic anhydride (TFAA, 5 equiv) to the acetamide in chamber A gas bubbles were observed within seconds. Hence the formation of trifluoroacetonitrile can easily be controlled by the speed of addition of TFAA. After complete addition the reaction was left for some time to ensure sufficient reaction time as TLC analysis is not easily done as the system is now under pressure. The chamber was then opened to release excess gas (on small scale) and the product in chamber B can be obtained by concentration and purification. The use of other bases than DBU, was also investigated. Similar to the synthesis of trichloroacetimidates K_2_CO_3_ was effective for the synthesis of β-acetimidates [26], whereas polystyrene-supported DBU [27] was found to be less effective and DBU was therefore the preferred catalyst.

The synthesis of perbenzylated α-glucosyl trifluoroacetimidate **3** proceeded in 52% yield on a preparative scale, with DBU as the base, which is comparable with the yields typically obtained in syntheses of trichloroacetimidates (Figure 2). When K_2_CO_3_ was used 44% (72% conversion) could be obtained with the β-product **5** as the major product (α/β = 32:68). Attempts to make the method column-free by filtering-off the base turned out to be unsuccessful as the reaction seemed to achieve an equilibrium, with some amount of hemiacetal left. Generally, it was observed that the trifluoroacetimidates were somewhat more sensitive to flash column chromatography, suggesting a higher reactivity compared to the trichloroacetimidates, as described by Schmidt [24]. The acetylated glucosyl trifluoroacetimidate **4** was synthesized in 66% yield (Figure 2). The slightly higher isolated yield is reflecting the higher stability of the product and hence an easier purification. The two-chamber method clearly works for the generation of trifluoroacetonitrile in a safe and easy manner. To expand the scope, we chose two other commercially available haloacetamides as precursors. Hence bromodifluoroacetamide and pyridine were dissolved in dichloromethane in chamber A followed by the addition of trifluoroacetic anhydride. This resulted in a clean transformation of the peracetylated glucose hemiacetal **2** in the other chamber (B), giving 73% isolated yield of the corresponding acetimidate **6** (Figure 2). Similarly, the chlorodifluoroacetimidate **7** was synthesized in 79% yield. From these reactions it is clear that the less reactive (disarmed) peracetylated glycosyl trifluoroacetimidates are more stable and hence easier to purify and store, whereas the more activated benzyl-protected analog **3** is unstable and decomposes in contact with silica unless the silica had been deactivated with triethylamine in the eluent. It was furthermore found that the best yields were obtained when using purification by “dry column vacuum chromatography” [28], which allowed separation of anomers. In the seminal paper by Schmidt [29] dichloroacetimidates were also synthesized. They were described as too unreactive to be viable alternatives to the trichloro analogs. The synthesis of difluoroacetimidates was therefore attempted, but in our hands, these proved even more unstable than the trifluoroacetimidates and were not possible to isolate by chromatography due to decomposition. However, the purity of these compounds, after filtration through a block of silica gel, was sufficient to be used in a subsequent reaction, hence demonstrating the advantage of using a two-chamber system compared to a one-pot setup, where excess reagents would be present in the crude.

**Figure 3:**
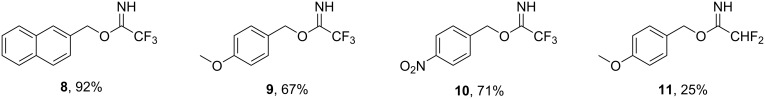
Synthesis of arylmethylene fluoroacetimidates using the two-chamber method.

As mentioned in the introduction, trifluoroacetonitrile is often used to synthesize a variety of heterocycles via the trifluoroacetimidate. We therefore decided to use the two-chamber method for the synthesis of a few non-carbohydrate acetimidates **8**–**11** (Figure 3). Hence a series of aryl alcohols with different substituents on the aromatic ring were synthesized and isolated in generally good yields. One exception was the *p*-methoxy derivative **11** which, as expected, was more reactive and hence more difficult to purify due to decomposition on silica gel. The *p*-methoxybenzyl trifluoroacetimidate (**9**) has been shown to be an effective regent for the acid-catalyzed benzylation of alcohols [12,30].

## Conclusion

In conclusion, we have developed a simple and safe methodology for the on-demand ex situ generation of stoichiometric amounts of gaseous haloacetonitriles in a closed system at room temperature. The methodology is safe, robust, and operationally simple on a laboratory scale. The method was exemplified by the synthesis of several glycosyl haloacetimidates and benzyl fluoroacetimidates, with different substituent patterns. The method allows for a larger substrate scope of halonitriles than earlier methods and is easily facilitated by low cost commercially available equipment.

## Supporting Information

File 1 Experimental section.

## Data Availability

All data that supports the findings of this study is available in the published article and/or the supporting information of this article.
