# Supplementary material for: Ex-situ generation of gaseous nitriles in two-chamber glassware for facile haloacetimidate synthesis
Source: Beilstein J Org Chem. 2025 Nov 7;21:2465–9. doi: 10.3762/bjoc.21.188 (PMC12599395; doi:10.3762/bjoc.21.188)
Supplement: File 1 — Experimental section. [file Beilstein_J_Org_Chem-21-2465-s001.pdf]

BEILSTEIN JOURNAL OF ORGANIC CHEMISTRY

Supporting Information

for

Ex-situ generation of gaseous nitriles in two-chamber glassware for facile haloacetimidate synthesis

Nikolai B. Akselvoll, Jonas T. Larsen and Christian M. Pedersen

Beilstein J. Org. Chem. **2025,** *21,* 2465–2469. doi:10.3762/bjoc.21.188

Experimental section

License and Terms: This is a supporting information file under the terms of the Creative Commons Attribution License (https://creativecommons.org/licenses/by/4.0). Please note that the reuse, redistribution and reproduction in particular requires that the author(s) and source are credited and that individual graphics may be subject to special legal provisions.
The license is subject to the *Beilstein Journal of Organic Chemistry* terms and conditions: (https://www.beilstein-journals.org/bjoc/terms)

Experimental section

General methods

The chemicals were directly applied as purchased without further purification. Dry solvents were from an Innovative Technology PS-MD-05 solvent system. All reactions were carried out in flame-dried glassware and under a nitrogen atmosphere unless otherwise stated. Plates used for thin-layer chromatography were silica gel (60 F_{254}) on aluminum sheets. The plates were first analyzed under UV light then stained in a 10% solution of H_2SO_4 in ethanol. Purification by flash chromatography was carried out with silica gel (40–63 μm). Purification with dry column vacuum chromatography was carried out with silica gel (15–40μm).

^{1}H NMR and ^{13}C NMR spectra were recorded at 298 K on a Bruker 500 MHz Ultra Shield Plus spectrograph equipped with a cryoprobe or a Bruker 500 MHz Ultra Shield Plus spectrograph equipped with an inverse cryoprobe. ^{19}F NMR was recorded at 300 K on a 500 MHZ Bruker instrument with a broadband observe probe. ^{1}H NMR spectra were recorded at 500 MHz, ^{13}C NMR at 126 MHz, and ^{19}F NMR at 470 MHz. The spectra were referenced to the non-deuterated solvent peaks in $CDCl_3$ (^{1}H: 7.26 ppm ^{13}C: 77.16 ppm), CD_3OD (^{1}H: 3.31 ppm ^{13}C: 49.00 ppm) or DMSO-d_6 (^{1}H: 4.79 ppm ^{13}C: 39.52 ppm). In ^{19}F NMR the spectrum is referenced to 1,3,5-tris(trifluoromethyl)benzene at −63.05 ppm in $CDCl_3$ and −63.79 ppm in CD_3OD. High resolution mass spectrometry (HRMS) was carried out on a Bruker SolariX XR7T ESI/MALDI-FT-ICR-MS using matrix-assisted laser desorption ionization (MALDI) with dithranol as the matrix.

General procedures for formation of trihaloacetimidates

All trihaloacetimidate formations are performed in a SyTracks 20 mL two chamber reaction vessel which consists of two reaction chambers that are connected by glass tubing.

Chamber A:

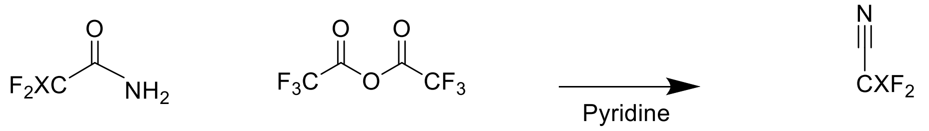

X = F, Cl, Br, H

Pg = Ac or Bn

Chamber B:

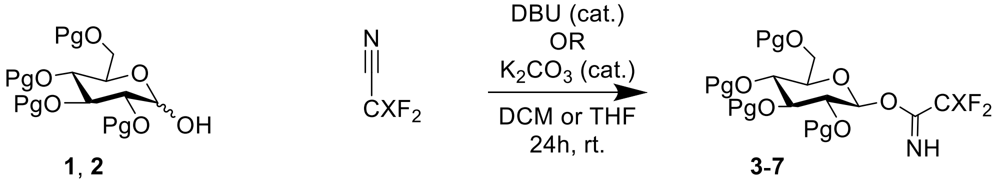

General procedure A:

2,2,2-Trifluoroacetamide (6 equiv) was added dissolved in pyridine (5 mL) in chamber A in a two-chamber reaction vessel. 2,3,4,6-Tetra-*O*-acetyl/benzyl-D-glucopyranose (500 mg scale, 1 equiv), K_2CO_3 (0.4 equiv or 1.1 equiv) and DCM or THF (5 mL) was added to chamber B. The reaction vessel was closed tightly and trifluoroacetic anhydride (5 equiv) was injected through a septum into reaction chamber A. After 24 h, the reaction vessel was opened inside the fume hood and solids in chamber B filtered off using a syringe filter. The remaining solution is evaporated onto silica and purified by flash column chromatography.

General procedure B:

2,2,2-Trifluoroacetamide (6 equiv) was added dissolved in pyridine (5 mL) in chamber A in a two-chamber reaction vessel. 2,3,4,6-Tetra-*O*-acetyl/benzyl-D-glucopyranose (500 mg scale, 1 equiv), DBU (3 drops) and DCM or THF (5 mL) was added to chamber B. The reaction vessel was closed tightly and trifluoroacetic anhydride (5 equiv) was injected through a septum into reaction chamber A. Before opening the reaction vessel, a dry column was packed and rinsed with 1% Et_3N in *n*-heptane followed by *n*-heptane. After 24 h, the reaction vessel was opened inside the fume hood, the contents transferred to the top of the dry column and purified by means of dry column vacuum chromatography [1].

***O*-(2,3,4,6-Tetra-*O*-benzyl-α-D-glucopyranosyl) trifluoroacetimidate (3-α)**

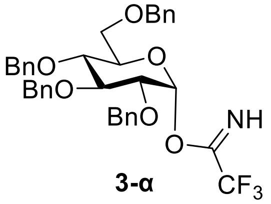

3-α

The synthesis and purification was performed in line with general procedure B.

Description: Colorless syrup.

Yield **52%**

A mixture of the α/ß anomers of the product can be acquired using general procedure A.

Yield **44%**, α/ß-ratio: 38/62

Data presented is in accordance with literature values[2]

^{1}H NMR (500 MHz, $CDCl_3$) δ 8.48 (s, 1H, **NH**), 7.35 – 7.27 (m, 18H, **Ph**), 7.15 (dd, *J* = 7.2, 2.3 Hz, 2H, **Ph**), 6.53 (d, *J* = 3.4 Hz, 1H, **H1**), 4.97 (d, *J* = 10.8 Hz, 1H, **PHCH$_2$**), 4.84 (dd, *J* = 10.8, 7.5 Hz, 2H, **PHCH$_2$**), 4.71 (d, *J* = 3.7 Hz, 2H, **PHCH$_2$**), 4.61 (d, *J* = 12.1 Hz, 1H, **PHCH$_2$**), 4.52 (d, *J* = 10.6 Hz, 1H, **PHCH$_2$**), 4.47 (d, *J* = 12.1 Hz, 1H, **PHCH$_2$**), 4.01 (t, *J* = 9.4 Hz, 1H, **H3**), 3.89 (dt, *J* = 10.1, 2.5 Hz, 1H, **H5**), 3.82 – 3.77 (m, 2H, **H4**, **H6$_A$**), 3.75 (dd, *J* = 9.6, 3.5 Hz, 1H, **H2**), 3.66 (dd, *J* = 11.0, 2.0 Hz, 1H, **H6$_B$**).

^{13}C NMR (126 MHz, $CDCl_3$) δ 138.74 (**Ph**), 138.13 (**Ph**), 137.93 (**Ph**), 128.58 (**Ph**), 128.54 (**Ph**), 128.15 (**Ph**), 128.10 (**Ph**), 128.06 (**Ph**), 127.98 (**Ph**), 127.91 (**Ph**), 127.87 (**Ph**), 127.78 (**Ph**), 94.11 (**C1**), 80.96 (**C3**), 79.64 (**C2**), 76.8 (**C4**), 75.87 (**PhCH$_2$**), 75.48 (**PhCH$_2$**), 73.65 (**PhCH$_2$**), 73.28 (**PhCH$_2$**), 73.10 (**C5**), 68.69 (**C6**).

The multiplet from the halomethyl carbon atom was not observed due to poor signal to noise ratio.

^{19}F NMR (470 MHz, $CDCl_3$) δ -74.57.

$R_{F\text{-}\alpha}$ = 0.51 (1:1 EtOAc:Hep) $R_{F\text{-}ß}$ = 0.40 (1:1 EtOAc:Hep)

***O*-(2,3,4,6-Tetra-*O*-acetyl-α-D-glucopyranosyl) trifluoroacetimidate (4)**

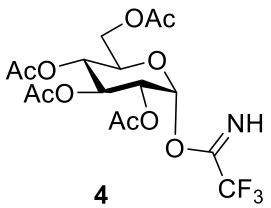

4

The synthesis and purification was performed in line with general procedure B.

Description: Colorless syrup.

Yield **50%**

A mixture of the α/ß anomers of the product can be acquired using general procedure A.

Yield in DCM **58%** α/ß-ratio: 16/84

Yield in THF **66%** α/ß-ratio: 25/75

Data presented is in accordance with literature values.[2]

^{1}H NMR (500 MHz, $CDCl_3$) δ 8.64 (s, 1H, **NH**), 6.58 (d, *J* = 3.7 Hz, 1H, **H1**), 5.51 (dd, *J* = 10.2, 9.5 Hz, 1H, **H3**), 5.18 (t, *J* = 9.8 Hz, 1H, **H4**), 5.13 (dd, *J* = 10.3, 3.7 Hz, 1H, **H2**), 4.28 (m, 1H, **$H6_A$**), 4.14 – 4.09 (m, 2H, **H5**, **$H6_B$**), 2.08 (s, 3H, **$AcCH_3$**), 2.05 (s, 3H, **$AcCH_3$**), 2.03 (s, 3H, **$AcCH_3$**), 2.02 (s, 3H, **$AcCH_3$**).

^{13}C NMR (126 MHz, $CDCl_3$) δ 170.68 (**C=O**), 170.15 (**C=O**), 169.95 (**C=O**), 169.59 (**C=O**), 91.98 (**C1**), 70.07, 69.86 (**C3**, **C5**), 69.05 (**C2**), 67.81 (**C4**), 61.40 (**C6**), 20.74 (2C, **$AcCH_3$**), 20.69 (**$AcCH_3$**), 20.54 (**$AcCH_3$**).

The multiplet from the halomethyl carbon atom was not observed due to poor signal to noise ratio

^{19}F NMR (470 MHz, $CDCl_3$) δ -74.57.

R_F: 0.58 (1:1 Hep:EtOAc)

***O*-(2,3,4,6-Tetra-*O*-acetyl-β-D-glucopyranosyl) trifluoroacetimidate (5)**

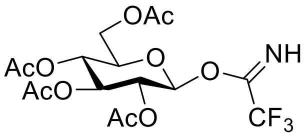

5

This was isolated in smaller amount when synthesizing the α-anomer by general procedure B.
Description: Whiter amorphous powder

^{1}H NMR (500 MHz, $CDCl_3$) δ 8.69 (s, 1H, **NH**), 5.88 (d, *J* = 7.7 Hz, 1H, **H1**), 5.25 (m, 2H, **H2, H3**), 5.17 (t, *J* = 9.4 Hz, 1H, **H4**), 4.29 (dd, *J* = 12.5, 4.4 Hz, 1H, **$H6_A$**), 4.14 (dd, *J* = 12.5, 2.4 Hz, 1H, **$H6_B$**), 3.89 (ddd, *J* = 10.0, 4.4, 2.4 Hz, 1H, **H5**), 2.07 (s, 3H, **$AcCH_3$**), 2.02 (s, 3H, **$AcCH_3$**), 2.02 (s, 3H, **$AcCH_3$**), 2.01 (s, 3H, **$AcCH_3$**).

^{13}C NMR (126 MHz, $CDCl_3$) δ 170.76 (**C=O**), 170.27 (**C=O**), 169.47 (**C=O**), 169.13 (**C=O**), 94.30 (**C1**), 72.87 (**C5**), 72.59 (**C2**), 70.19 (**C3**), 67.83 (**C4**), 61.51 (**C6**), 20.78 (**$AcCH_3$**), 20.68 (2C, **$AcCH_3$)**, 20.46 (**$AcCH_3$**).
The multiplet from the halomethyl carbon atom was not observed due to poor signal to noise ratio

^{19}F NMR (470 MHz, $CDCl_3$) δ -74.81.

R_F: 0.45 (1:1 Hep:EtOAc)

***O*-(2,3,4,6-Tetra-*O*-acetyl-α-D-glucopyranosyl) chlorodifluoroacetimidate (7)**

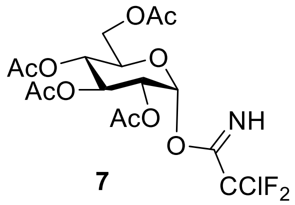

7

The synthesis and purification was performed in line with general procedure B.

Description: Colorless syrup.

Yield **79%**

Data presented is in accordance with literature values.[2]

^{1}H NMR (500 MHz, $CDCl_3$) δ 8.47 (s, 1H, **NH**), 6.56 (d, *J* = 3.7 Hz, 1H, **H1**), 5.52 (dd, *J* = 10.2, 9.5 Hz, 1H, **H3**), 5.17 (t, *J* = 9.8 Hz, 1H, **H4**), 5.13 (dd, *J* = 10.2, 3.7 Hz, 1H, **H2**), 4.27 (dd, *J* = 12.2, 3.8 Hz, 1H, **$H6_A$**), 4.17 – 4.13 (m, 1H, **H5**), 4.12 – 4.08 (m, 1H **$H6_B$**), 2.07 (s, 3H, **$AcCH_3$**), 2.04 (s, 3H, **$AcCH_3$**), 2.02 (s, 3H, **$AcCH_3$**), 2.01 (s, 3H, **$AcCH_3$**).

^{13}C NMR (126 MHz, $CDCl_3$) δ 170.68 (**C=O**), 170.13 (**C=O**), 169.94 (**C=O**), 169.60 (**C=O**), 92.08 (**C1**), 70.08, 69.87 (**C3**, **C5**), 69.55 (**C2**), 67.82 (**C4**), 61.42 (**C6**), 20.76 (2C, **$AcCH_3$**), 20.67 (**$AcCH_3$**), 20.51 (**$AcCH_3$**).

The multiplet from the halomethyl carbon atom was not observed due to poor signal to noise ratio

^{19}F NMR (470 MHz, $CDCl_3$) δ -62.36, -64.35.

HRMS MALDI-TOF (M+Na$^+$) $C_{16}H_{20}ClF_2NNaO_{10}^+$ for calculated 482.06360 found 482.06208 (3.2 ppm)

R_F-α: 0.53 (1:1 Hep:EtOAc) R_F-β: 0.32 (50:50 Hep:EtOAc)

_O_-(2,3,4,6-Tetra-_O_-acetyl-α-D-glucopyranosyl) bromodifluoroacetimidate (6)

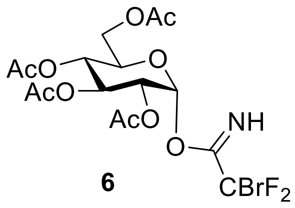

6

The synthesis and purification was performed in line with general procedure B.
Description: Colorless syrup.
Yield **73%**

^{1}H NMR (500 MHz, $CDCl_3$) δ 8.47 (s, 1H, **NH**), 6.47 (d, *J* = 3.8 Hz, 1H, **H1**), 5.44 (t, *J* = 9.9 Hz, 1H, **H3**), 5.08 (t, *J* = 9.9 Hz, 1H, **H4**), 5.04 (dd, *J* = 10.2, 3.7 Hz, 1H, **H2**), 4.18 (dd, *J* = 12.5, 4.1 Hz, 1H, **$H6_A$**), 4.09 (ddd, *J* = 10.3, 4.1, 2.2 Hz, 1H, **H5**), 4.02 (dd, *J* = 12.4, 2.2 Hz, 1H, **$H6_B$**), 1.97 (s, 3H, **$AcCH_3$**), 1.95 (s, 3H, **$AcCH_3$**), 1.93 (s, 3H, **$AcCH_3$**), 1.92 (s, 3H, **$AcCH_3$**).

^{13}C NMR (126 MHz, $CDCl_3$) δ 170.39 (**C=O**), 169.87 (**C=O**), 169.68 (**C=O**), 169.37 (**C=O**), 92.12 (**C1**), 69.94, 69.67 (**C3**, **C5**), 69.37 (**C2**), 67.62 (**C4**), 61.23 (**C6**), 20.50 (2C, **$AcCH_3$**), 20.42 (**$AcCH_3$**), 20.26 (**$AcCH_3$**).
The multiplet from the halomethyl carbon atom was not observed due to poor signal to noise ratio

^{19}F NMR (470 MHz, $CDCl_3$) δ -58.91.

HRMS MALDI-TOF (M+Na$^+$) $C_{16}H_{20}BrF_2NNaO_{10}^+$ for calculated 526.01309 found 526.01146 (3.1 ppm)

R_F-α = 0.58 (1:1 EtOAc:Hep) **R_F-β** = 0.45 (1:1 EtOAc:Hep)

2,3,4,6-Tetra-*O*-acetyl-D-glucopyranose (2)

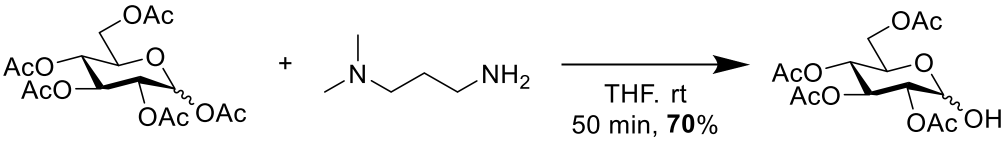

Following a procedure presented in literature [4], a solution of 1,2,3,4,6-penta-*O*-acetyl-D-glucopyranose (4.01g, 10.3 mmol) and DMAPA (6.45 mL, 51.2 mmol, 5 equiv) in THF (50 mL) was stirred at rt for 80 min, when TLC analysis revealed full conversion. The reaction mixture was diluted with CH_2Cl_2 (50 mL) and washed twice with 1 M HCl (2 × 25 mL) and once with brine (25 mL). The aqueous phase was washed once with CH_2Cl_2. The organic phases were combined and dried over $MgSO_4$, filtered, and evaporated to dryness in vacuo, yielding the product as a colorless syrup (3.19g, 9.17 mmol, **90%**).

NMR data of the product is in accordance with literature.[5]

R_F-α = 0.34 (1:1 EtOAc: Hep) **α/β**: 70:30

^{1}H NMR (500 MHz, CDCl3) δ 5.53 (dd, *J* = 10.2, 9.4 Hz, 1H, **H3α**), 5.46 (d, *J* = 3.6 Hz, 1H, **H1α**), 5.08 (t, *J* = 9.7, 1H, **H4α**), 4.90 (dd, *J* = 10.2, 3.6 Hz, 1H, **H2α**), 4.28-4.21 (m, 2H, **H5α**, **$H6_A$α**), 4.16-4.11 (m, 1H, **$H6_B$α**), 3.74 (m, 1H, **-OHα**), 2.09 (s, 3H, **AcO**), 2.08 (s, 3H, **AcO**), 2.03 (s, 3H, **AcO**), 2.01 (s, 3H, **AcO**).

^{13}C NMR (126 MHz, $CDCl_3$) δ 171.02 (**C=O**), 170.97 (**C=O**), 170.91 (**C=O**), 170.84 (**C=O**), 170.34 (**C=O**), 170.30 (**C=O**), 169.81 (**C=O**), 169.67 (**C=O**),
95.70 (**C1β**), 89.58 (**C1α**), 73.36 (**C2β**), 73.31, 72.34 (**C3β**, **C4β**), 72.21 (**C2α**), 69.99 (**C3α**), 68.62 (**C4α**), 68.56 (**C5β**), 68.10 (**C6β**), 67.35 (**C5α**), 62.10 (**C6α**), 25.72, 20.9 - 20.7 (**$AcCH_3$ x 8**).

***O*-(2,3,4,6-Tetra-*O*-acetyl-D-glucopyranosyl) difluoroacetimidate (S1)**

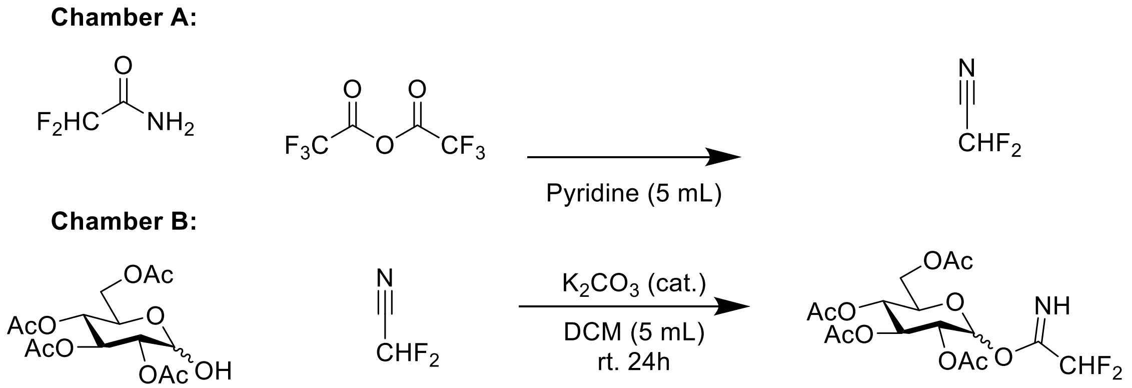

2,2-Difluoroacetamide (830 mg, 8.73 mmol, 6 equiv) was added dissolved in pyridine (5 mL) in chamber A in a two-chamber reaction vessel. 2,3,4,6-Tetra-*O*-acetyl-D-glucopyranose (500 mg, 1.44 mmol), K_2CO_3 (≈ 80 mg, 0.58 mmol, 0.4 equiv) and DCM (5 mL) was added to chamber B. The reaction vessel was closed tightly and TFAA (1.0 mL, 7.19 mmol, 5 equiv) was injected through a septum into reaction chamber A. After 22 h, reaction chamber A was put in a 35 °C water bath. After 24 h the reaction vessel was removed from heating and opened. The crude was evaporated onto silica and attempted to be purified by flash chromatography. The resulting colorless syrup contained mainly the product.

^{1}H NMR (500 MHz, $CDCl_3$) δ 8.47 (t, *J* = 2.1 Hz, 1H, **NH**), 6.56 (d, *J* = 3.7 Hz, 1H, **H1**), 5.93 (t, *J* = 54.6 Hz, 1H, **CF_2H**), 5.49 (dd, *J* = 10.3, 9.5 Hz, 1H, **H3**), 5.15 (t, *J* = 9.8 Hz, 1H, **H4**), 5.11 (dd, *J* = 10.3, 3.7 Hz, 1H, **H2**), 4.26 – 4.22 (m, 1H, **$H6_A$**), 4.10 – 4.06 (m, 2H, **H5**, **$H6_B$**), 2.06 (s, 3H, **$AcCH_3$**), 2.02 (s, 3H, **$AcCH_3$**), 2.01 (s, 3H, **$AcCH_3$**), 1.99 (s, 3H, **$AcCH_3$**).

^{13}C NMR (126 MHz, $CDCl_3$) δ 170.73 (**C=O**), 170.26 (**C=O**), 169.88 (**C=O**), 169.55 (**C=O**), 106.15 (t, *J* = 243.6 Hz, **CF_2H**), 90.94 (**C1**), 69.89, 69.74, 69.45, 67.88, 61.48, 20.75 (**$AcCH_3$**), 20.73 (**$AcCH_3$**), 20.64 (**$AcCH_3$**), 20.54 (**$AcCH_3$**).

R_F-α = 0.45 (2:1 EtOAc:Hep) **R_F-β** = 0.38 (2:1 EtOAc:Hep)

Synthesis of naphthalen-2-ylmethyl 2,2,2-trifluoroacetimidate (8)

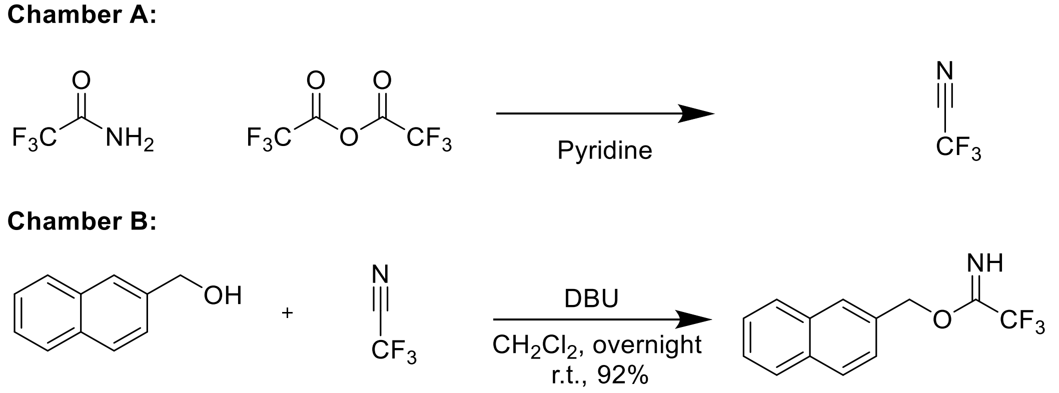

2,2,2-Trifluoroacetamide (6 equiv) was dissolved in pyridine (5 mL) in chamber A in a two-chamber reaction vessel. 2-Naphtalenemethanol (158 mg, 1.00 mmol, 1 equiv) and DBU (approx. 30 mg) was dissolved in DCM (4 mL) in chamber B. The reaction vessel was closed tightly and trifluoroacetic anhydride (5 equiv) was injected through a septum into reaction chamber A. After opening the reaction vessel, the reaction mixture was filtered through a cotton plug and reaction chamber B was washed with DCM (3 × 5 mL). The solvent was removed in vacuo and the crude product was purified by flash column chromatography (10:1 heptane (1% Et_3N)/ethyl acetate), yielding the product as a white powder (232 mg, 0.916 mmol, 92%).

^{1}H NMR (500 MHz, $CDCl_3$) δ 8.24 (s, 1H, **NH**), 7.81 – 7.77 (m, 4H, **ArH**), 7.45 – 7.41 (m, 3H, **ArH**), 5.40 (s, 2H, **CH_2**).

^{13}C NMR (126 MHz, $CDCl_3$) δ 133.40 (**Ar**), 128.64 (**Ar**), 128.19 (**Ar**), 127.90 (**Ar**), 127.52 (**Ar**), 126.58 (**Ar**), 126.55 (**Ar**), 125.73 (**Ar**), 69.56 (**CH_2**).

2 carbon signals from the naphthalene and the **C**F3 and **C**=N were not observed due to splitting giving low intensity.

Synthesis of 4-methoxybenzyl 2,2,2-trifluoroacetimidate (9)

Chamber A:

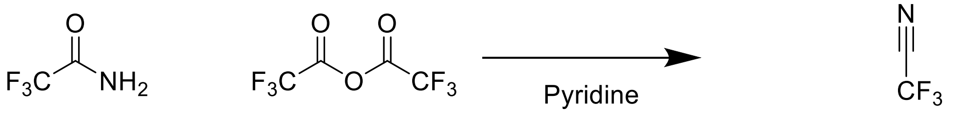

Chamber B:

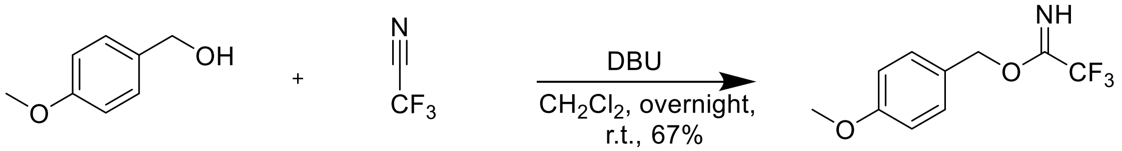

2,2,2-Trifluoroacetamide (6 equiv) was dissolved in pyridine (5 mL) in chamber A in a two-chamber reaction vessel. 4-Methoxybenzyl alcohol (222.0 mg, 0.200 mL, 1.61 mmol) and DBU (approx. 30 mg) was dissolved in DCM (4 mL) in chamber B. The reaction vessel was closed tightly and trifluoroacetic anhydride (5 equiv) was injected through a septum into reaction chamber A. After opening the reaction vessel, the reaction mixture was filtered through a cotton plug and reaction chamber B was washed with DCM (3 × 5 mL). The solvent was removed in vacuo and the crude product was purified by flash column chromatography (10:1 heptane (1% Et_3N)/ethyl acetate), yielding the product as a colorless oil (251.0 mg, 1.08 mmol, **67%**).

^{1}H NMR (500 MHz, $CDCl_3$) δ 8.26 (s, 1H, **NH**), 7.36 (d, *J* = 8.8 Hz, 2H, **ArH**), 6.92 (d, *J* = 8.7 Hz, 2H, **ArH**), 5.25 (s, 2H, **CH_2**), 3.82 (s, 3H, **CH_3**).

^{13}C NMR (126 MHz, $CDCl_3$) δ 160.0 (**Ar**), 158.0 (q, *J* = 37.9 Hz, **C=N**), 130.21 (**Ar**), 127.1 (**Ar**), 115.7 (q, *J* = 280.4 Hz, **CF_3**), 114.1 (**Ar**), 69.3 (**CH_2**), 55.4 (**CH_3**).

Synthesis of 4-nitrobenzyl 2,2,2-trifluoroacetimidate (10)

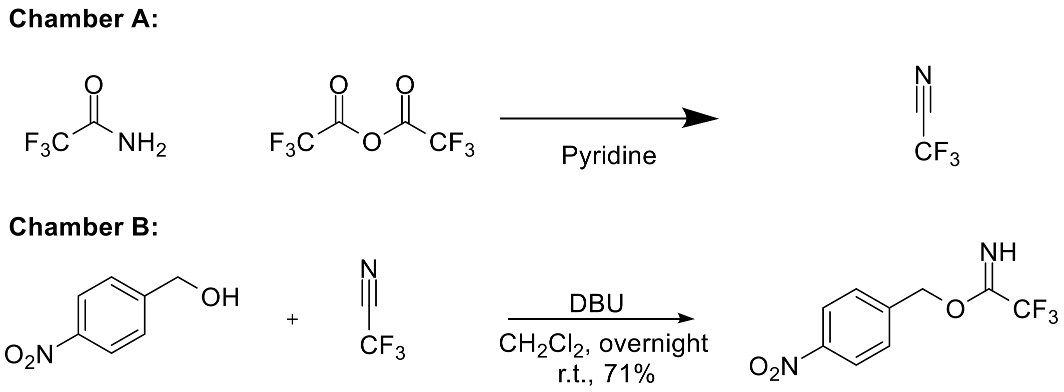

2,2,2-Trifluoroacetamide (6 equiv) was dissolved in pyridine (5 mL) in chamber A in a two-chamber reaction vessel. 4-Nitrobenzyl alcohol (153 mg, 1.00 mmol) and DBU (approx. 30 mg) was dissolved in DCM (4 mL) in chamber B. The reaction vessel was closed tightly and trifluoroacetic anhydride (5 equiv) was injected through a septum into reaction chamber A. After opening the reaction vessel, the reaction mixture was filtered through a cotton plug and reaction chamber B was washed with DCM (3 × 5 mL). The solvent was removed in vacuo and the crude product was purified by flash column chromatography (9:1 heptane (1% Et_3N)/ethyl acetate), yielding the product as a white powder (176 mg, 0.708 mmol, **71%**).

^{1}H NMR (500 MHz, $CDCl_3$) δ 8.33 (s, 1H, **NH**), 8.19 (d, *J* = 8.7 Hz, 2H, **ArH**), 7.50 (d, *J* = 8.8 Hz, 2H, **ArH**), 5.34 (s, 2H, **CH_2**).

^{13}C NMR (126 MHz, $CDCl_3$) δ 142.20 (**Ar**), 128.36 (**Ar**), 124.03 (**Ar**), 67.72 (**CH_2**).

The ^{13}C-NMR peaks of CF_3 and C=N were not observed due to splitting.

Synthesis of 4-methoxybenzyl 2,2-difluoroacetimidate (11)

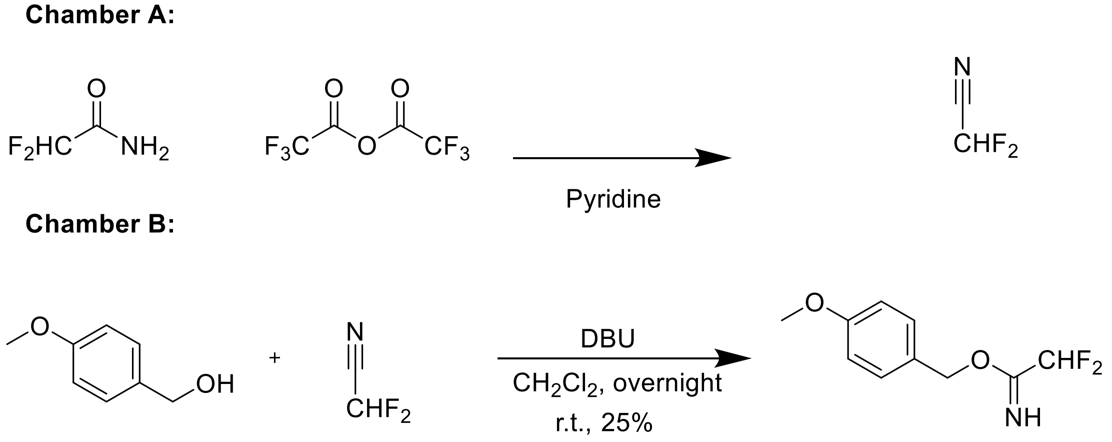

2,2-Difluoroacetamide (618 mg, 6.51 mmol, 3 equiv) was dissolved in pyridine (5 mL) in chamber A in a two-chamber reaction vessel. 4-Methoxybenzyl alcohol (300 mg, 2.17 mmol, 0.270 mL, 1 equiv), DBU (approx. 30. mg) and DCM (7 mL) was added to chamber B. The reaction vessel was closed tightly and trifluoroacetic anhydride (5 equiv) was injected through a septum into reaction chamber A. After opening the reaction vessel, the reaction mixture was filtered through a cotton plug and reaction chamber B was washed with DCM (3 × 5 mL). The solvent was removed in vacuo and the crude product was purified by flash column chromatography (9:1 heptane (1% Et_3N)/ethyl acetate), yielding the product as a colorless oil (188 mg, 0.549 mmol, **25%**).

^{1}H NMR (500 MHz, $CDCl_3$) δ 8.08 (s, 1H, **NH**), 7.34 (d, *J* = 8.4 Hz, 2H, **ArH**), 6.91 (d, *J* = 8.0 Hz, 2H, **ArH**), 5.84 (td, *J* = 54.9, 0.9 Hz, 1H, **CHF_2**), 5.21 (s, 2H, **CH_2**), 3.82 (s, 3H, **CH_3**).

^{13}C NMR (126 MHz, $CDCl_3$) δ 163.19 (t, *J* = 27.9 Hz, **C=N**), 159.91 (**Ar**), 130.6 (**Ar**), 130.1 (**Ar**), 114.0 (**Ar**), 106.7 (t, *J* = 243.0 Hz, **CHF_2**), 68.4 (**CH_2**), 55.3 (**CH_3**).

^{19}F NMR (470 MHz, $CDCl_3$) δ -124 (d, *J* = 50.9 Hz).

References:

[1] Pedersen, D., Rosenbohm, C. “Dry Column Vacuum Chromatography” *Synthesis* **2004**, *2001*, s-2001-18722.

[2] Nakajima, N.; Saito, M.; Kudo, M.; Ubukata, M. “Allyl, epoxy and glycosyl perfluoroimidates. One-pot preparation and reaction” *Tetrahedron* **2002**, *58*, 3579–3588.

[3] Schmidt, R. R.; Michel, J.; Roos, M. “Glycosylimidate, 12 Direkte Synthese von O-α- und O-β-Glycosyl-imidaten” *Liebigs Annalen der Chemie* **1984**, *1984*, 1343–1357.

[4] Andersen, S. M.; Heuckendorff, M.; Jensen, H. H. “3-(Dimethylamino)-1-propylamine: A cheap and versatile reagent for removal of byproducts in carbohydrate chemistry” *Organic Letters* **2015**, *17*, 944–947.

[5] Rønne Kristensen, B.; Pedersen, C. M. “Self□Promoted N□Glycosylation: Extended Substrate Scope and Substituent Effects” *Eur J Org Chem* **2023**, *26*, e202300213.

NMR spectra of characterized compounds

O-(2,3,4,6-Tetra-*O*-benzyl-α-D-glucopyranosyl) trifluoroacetimidate **3-α**

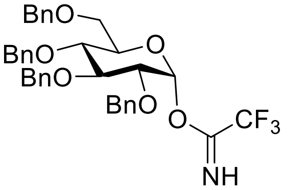

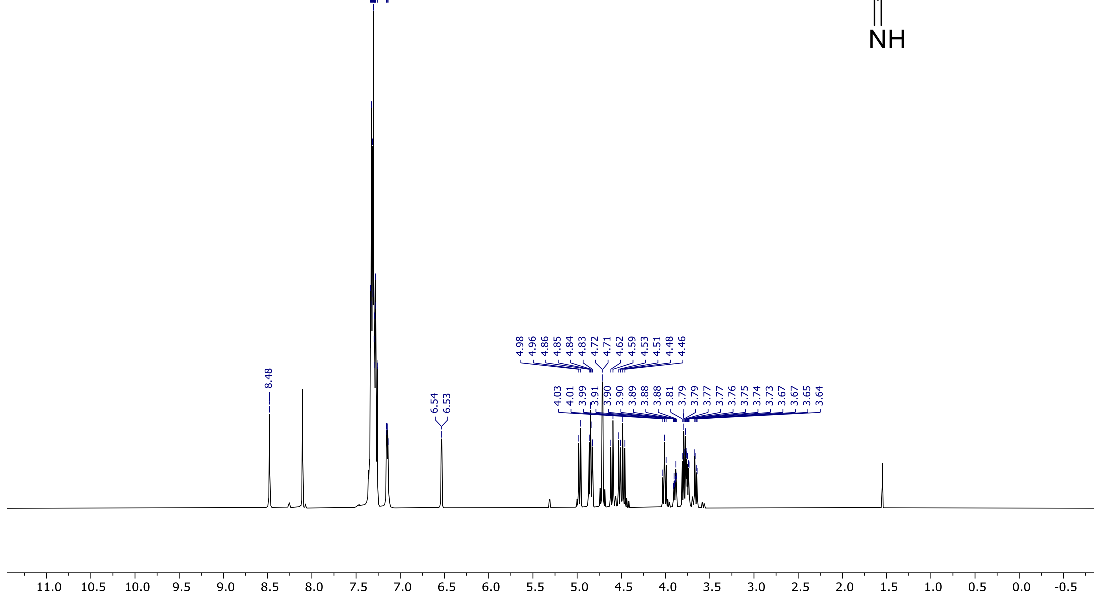

^{1}H NMR (500 MHz) in $CDCl_3$

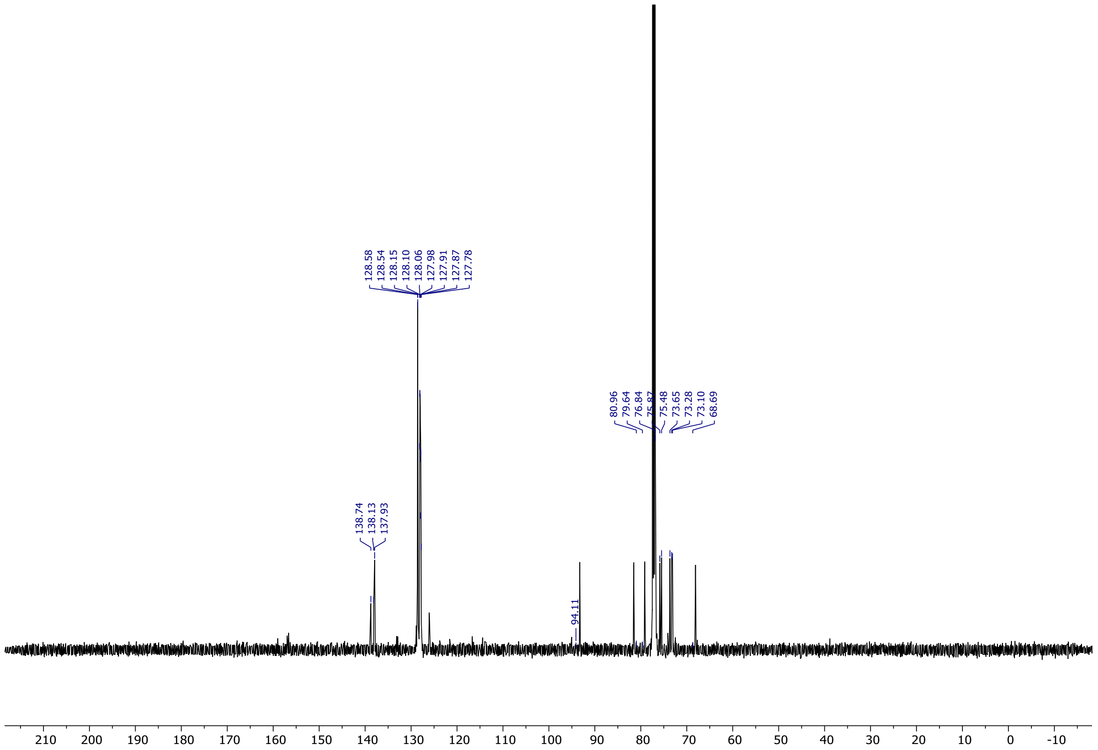

^{13}C NMR (126 MHz) in $CDCl_3$

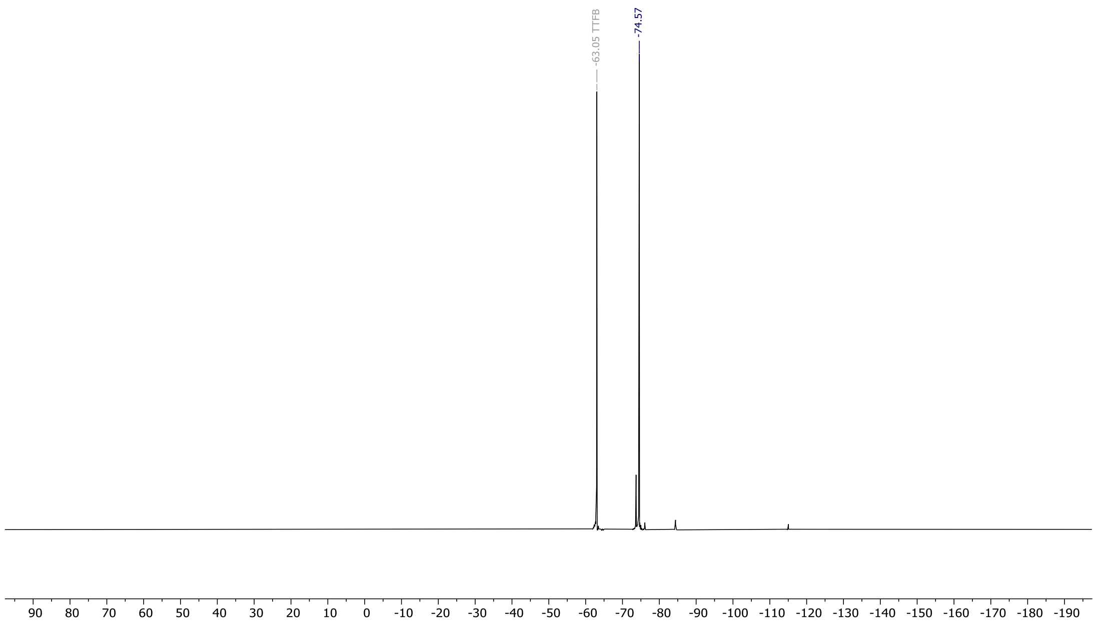

^{19}F NMR (470 MHz) in $CDCl_3$ with TTFB as reference

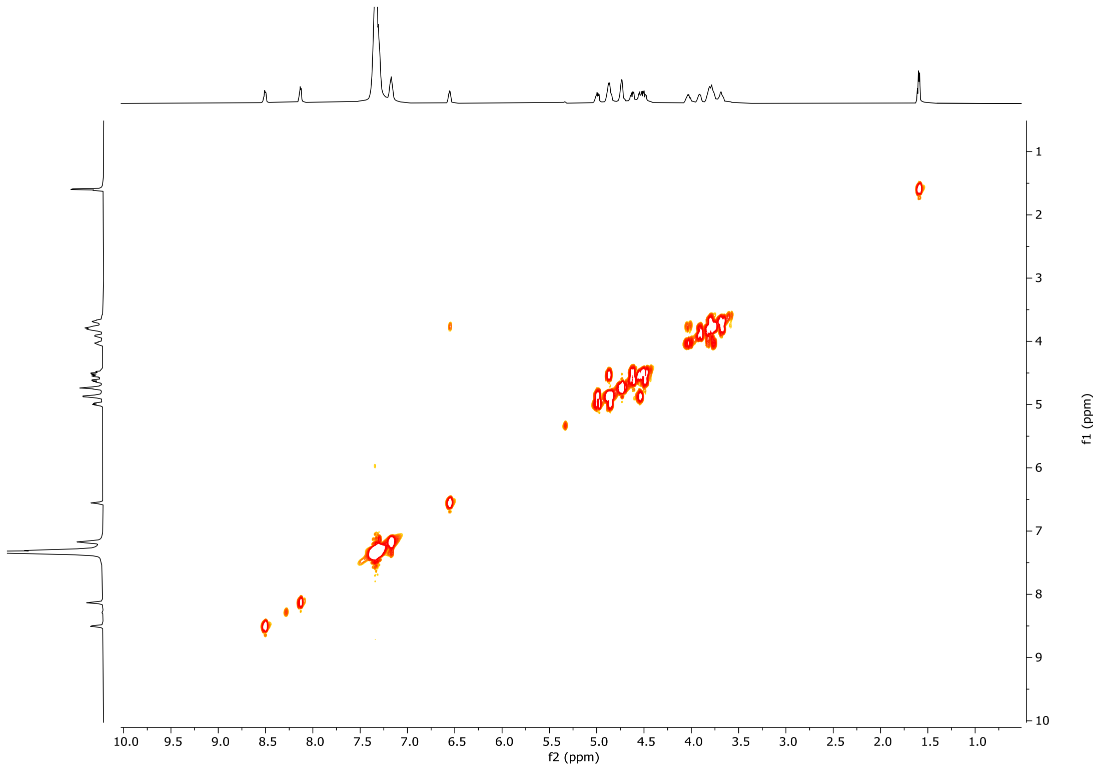

COSY (500 MHz) in $CDCl_3$

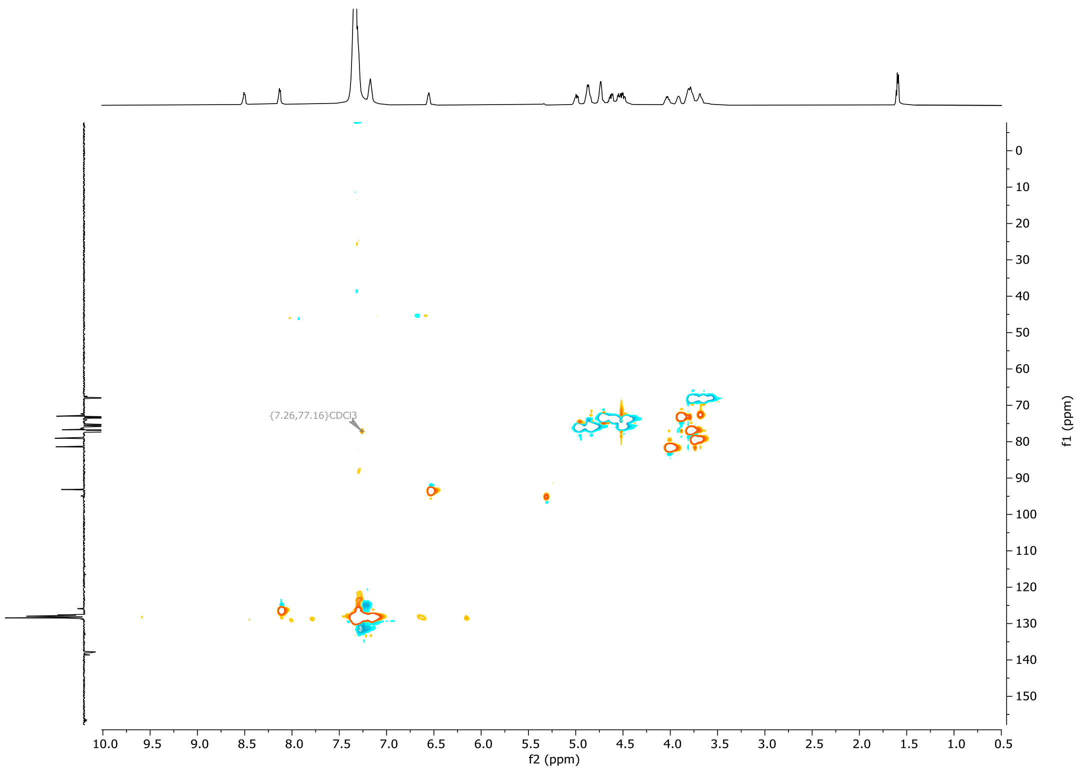

HSQC (500 MHz, 126 MHz) in $CDCl_3$

O-(2,3,4,6-Tetra-*O*-acetyl-α-D-glucopyranosyl) trifluoroacetimidate **2-α**

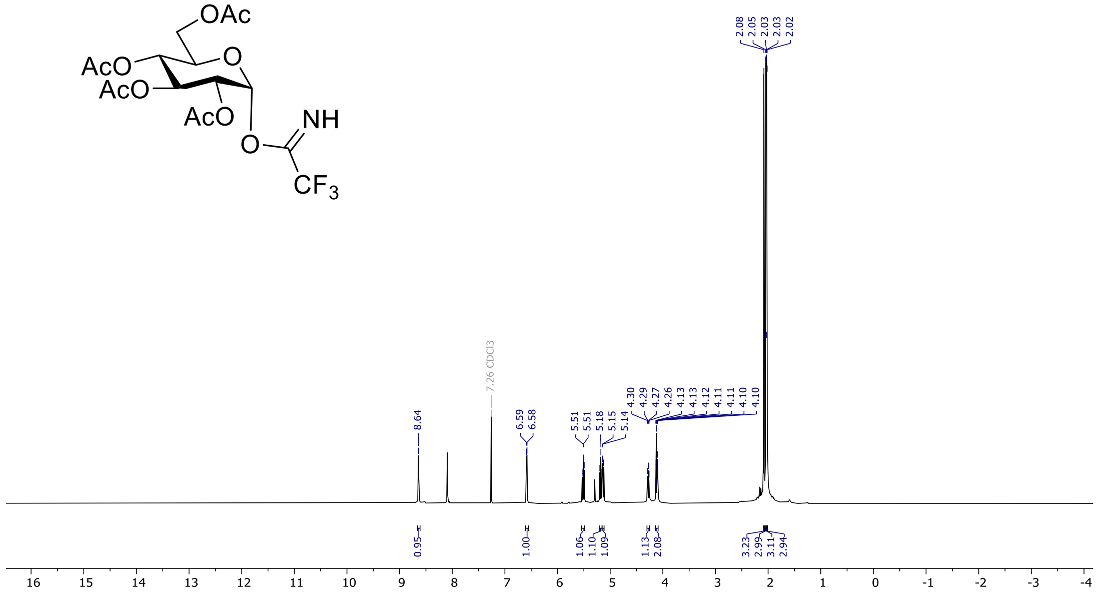

^{1}H NMR (500 MHz) in $CDCl_3$

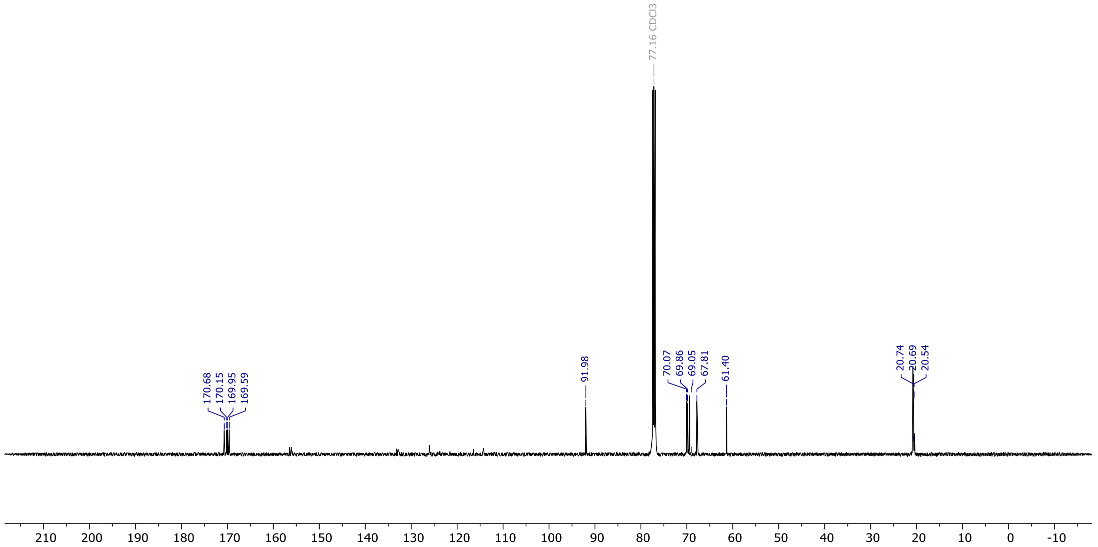

^{13}C NMR (126 MHz) in $CDCl_3$

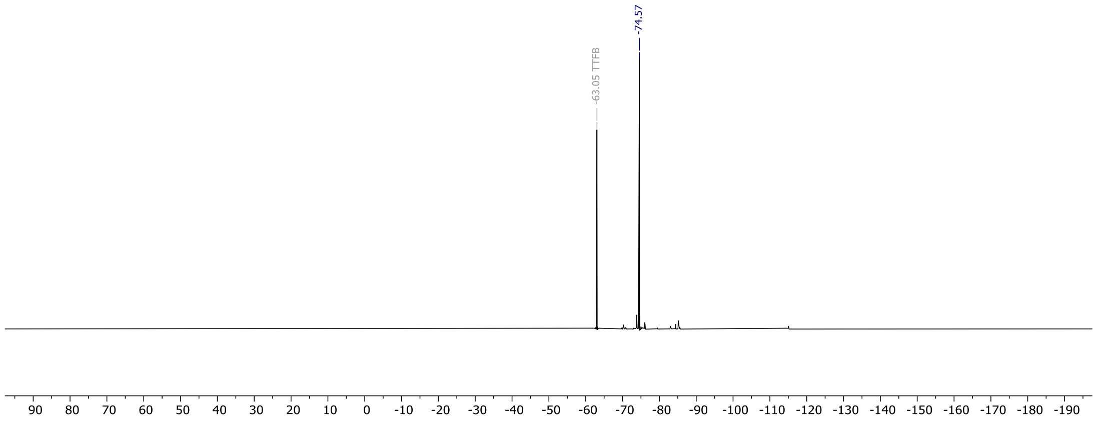

^{19}F NMR (470 MHz) in $CDCl_3$ with TTFB as reference

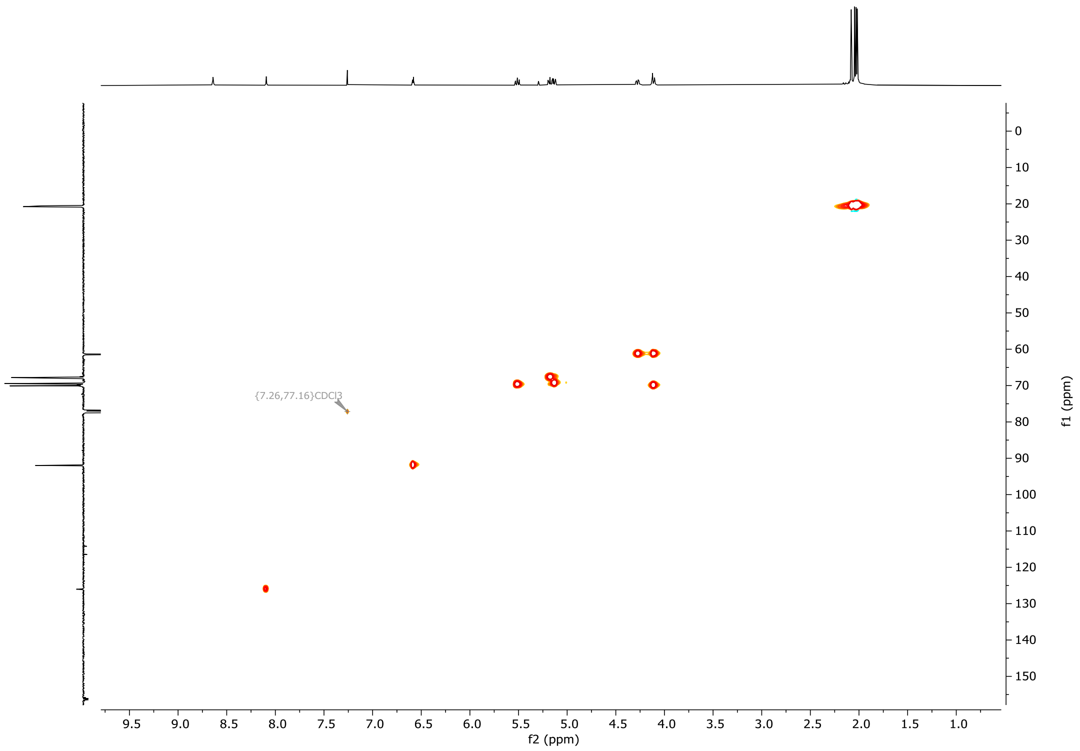

HSQC (500 MHz, 126 MHz) in $CDCl_3$

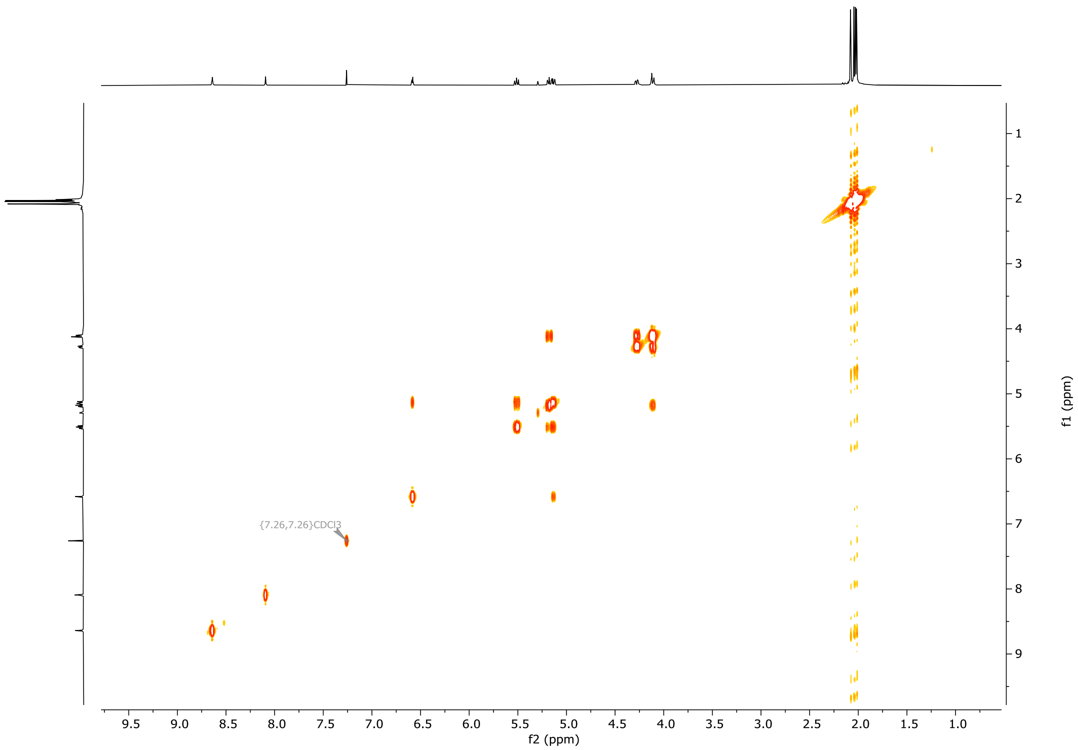

COSY (500 MHz) in $CDCl_3$

O-(2,3,4,6-Tetra-*O*-acetyl-β-D-glucopyranosyl) trifluoroacetimidate **2-β**

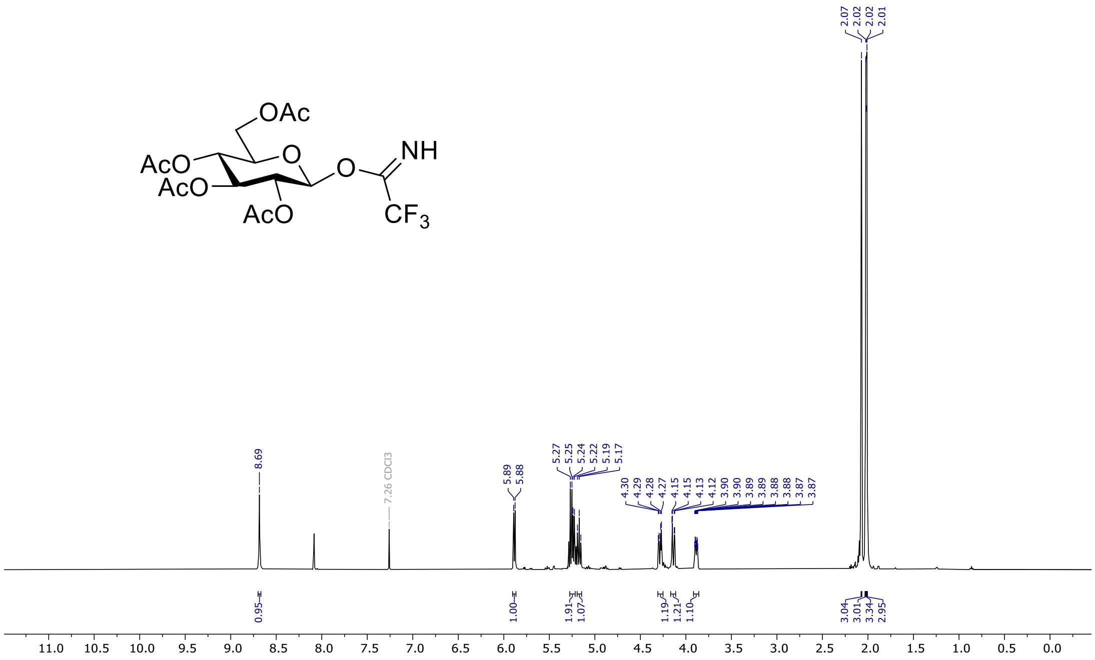

^{1}H NMR (500 MHz) in $CDCl_3$

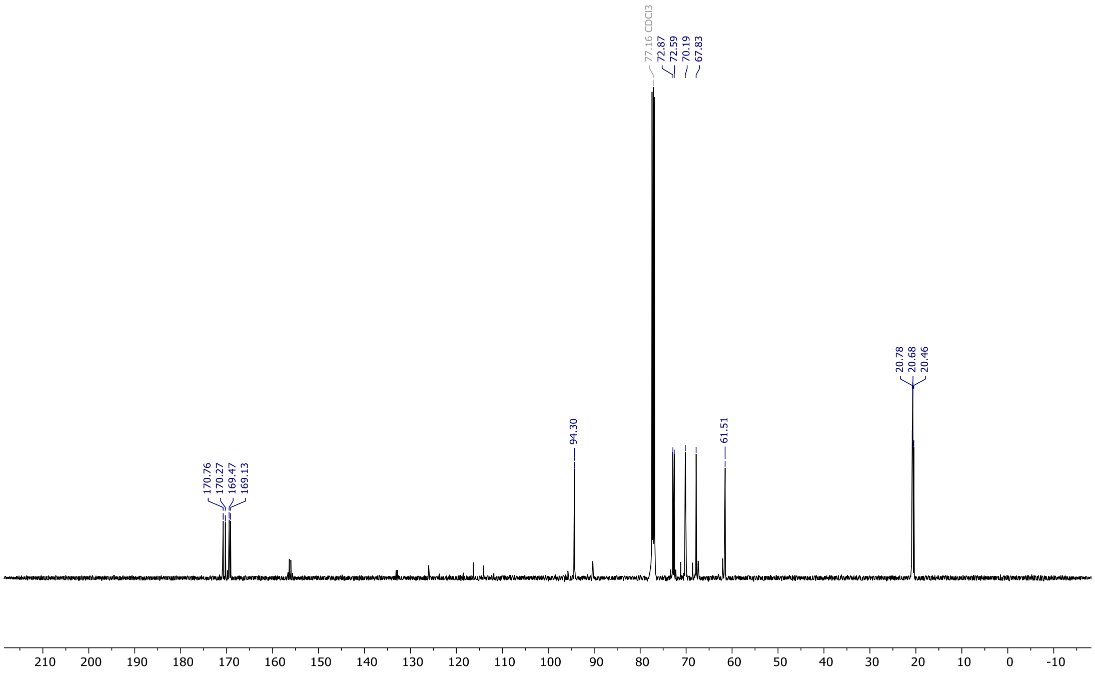

^{13}C NMR spectrum (126 MHz) in $CDCl_3$

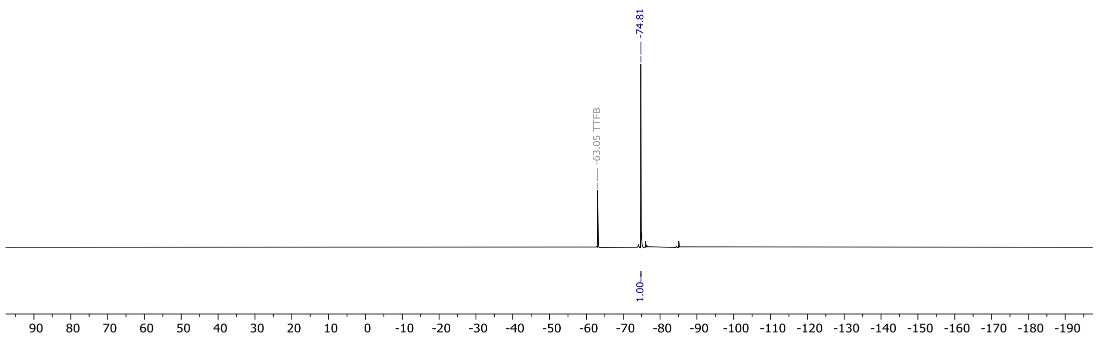

^{19}F NMR spectrum (470 MHz) in $CDCl_3$ with TTFB as standard

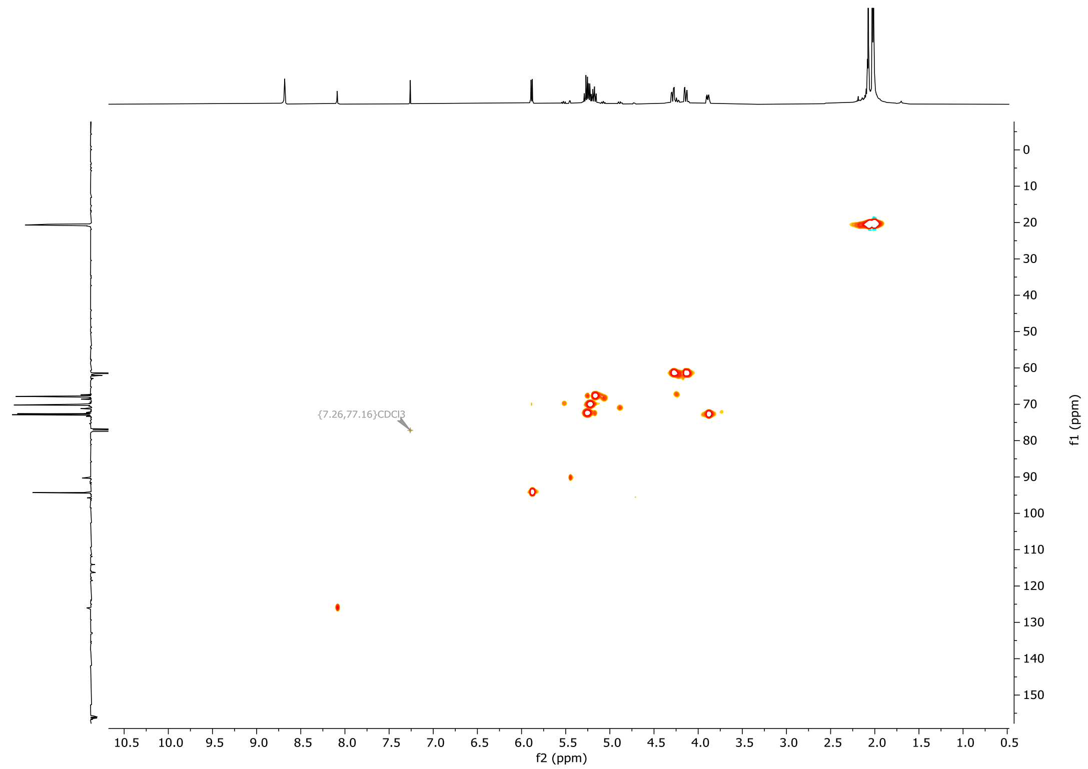

HSQC (500 MHz, 126 MHz) in $CDCl_3$

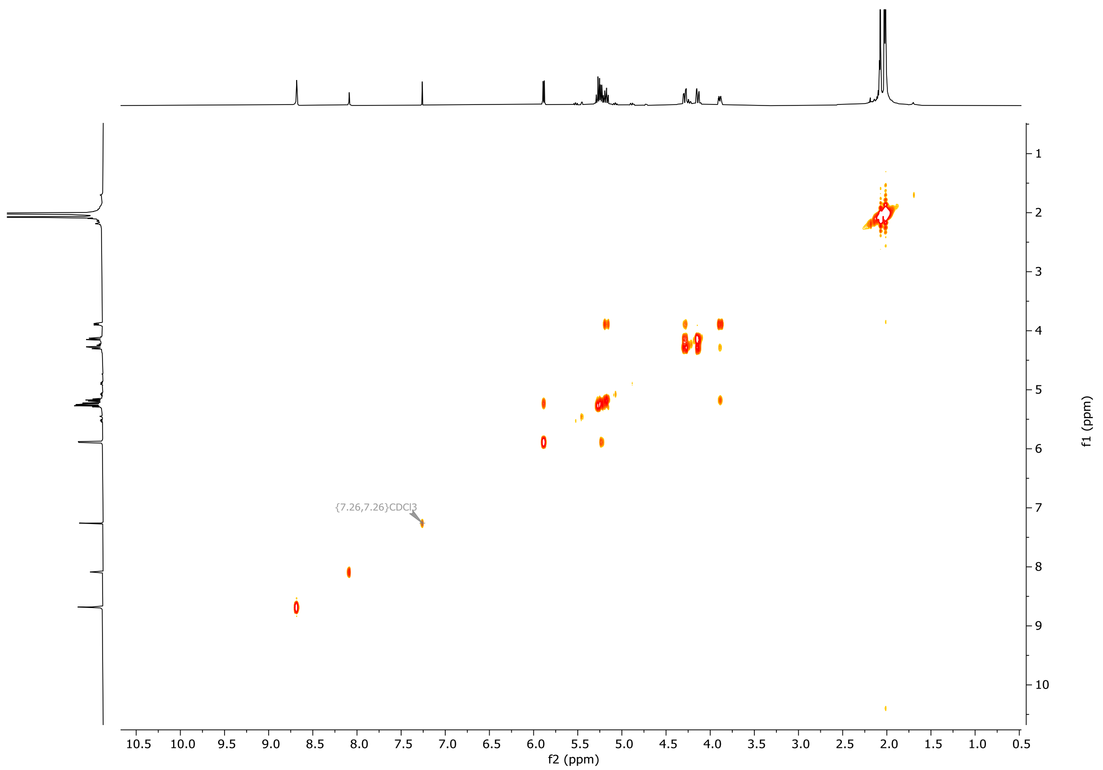

COSY (500 MHz) in $CDCl_3$

O-(2,3,4,6-Tetra-*O*-acetyl-α-D-glucopyranosyl) chlorodifluoroacetimidate **5-α**

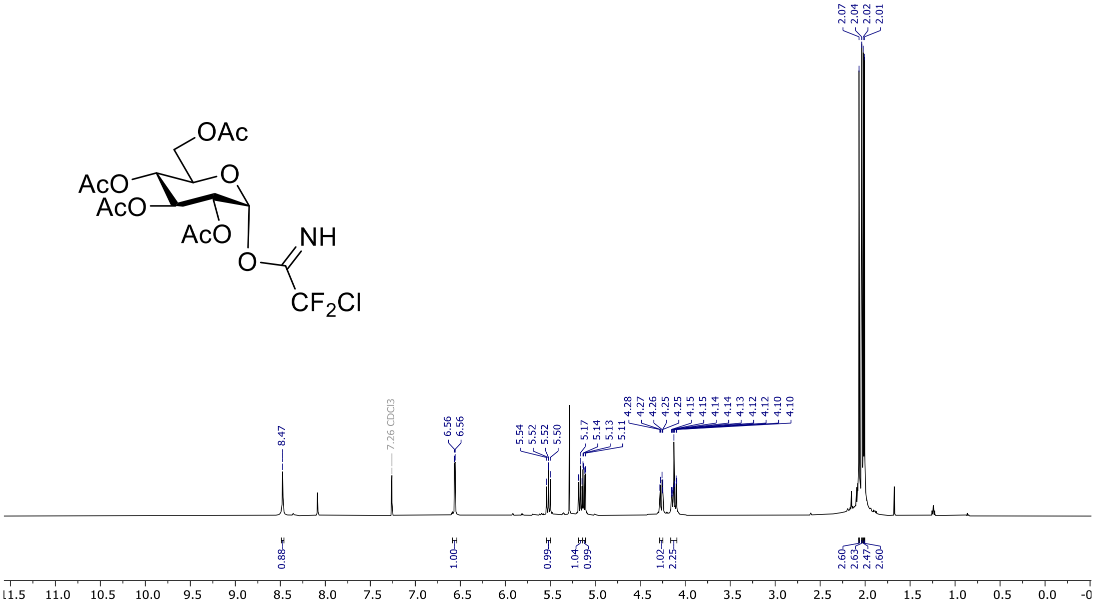

^{1}H NMR (500 MHz)) in $CDCl_3$

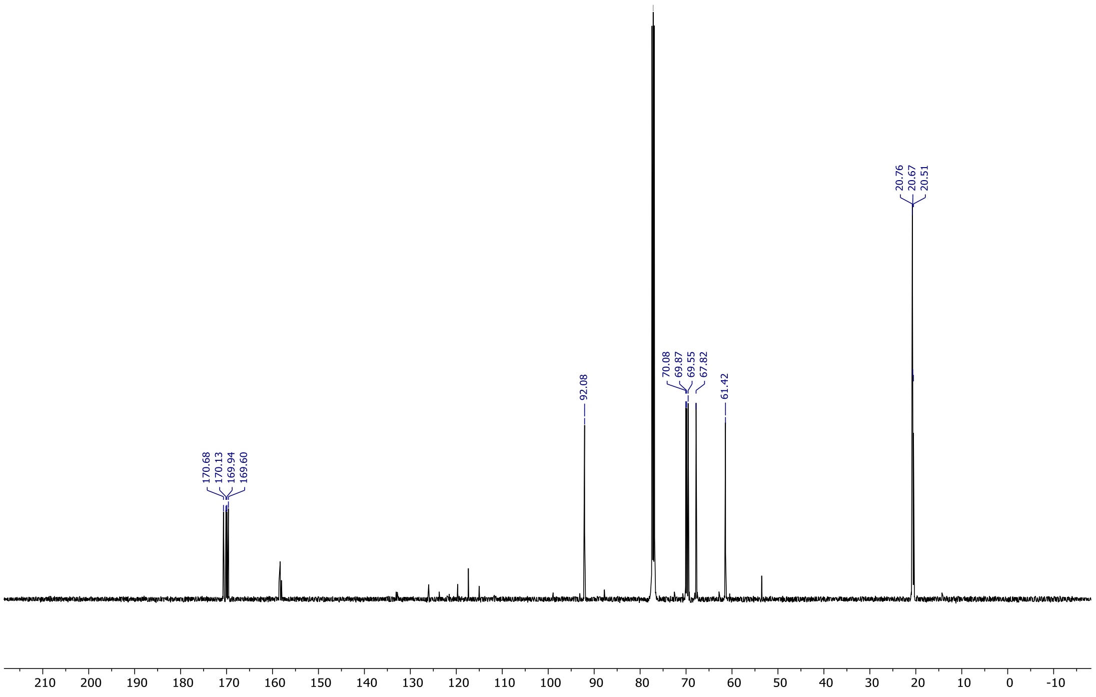

^{13}C NMR (126 MHz) in $CDCl_3$

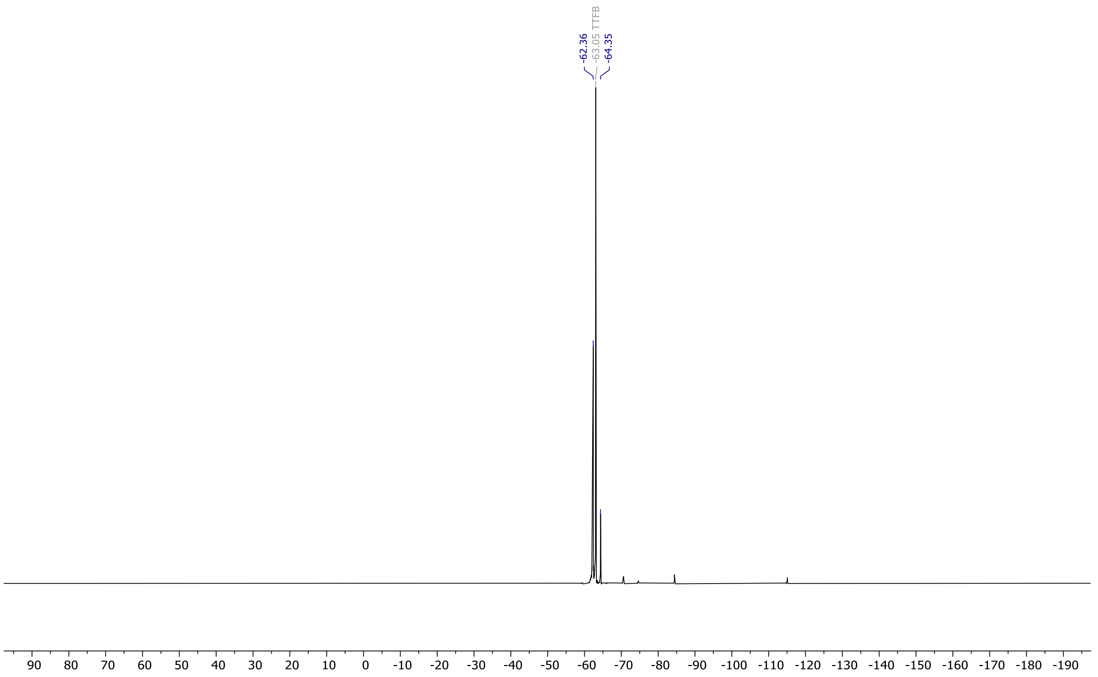

^{19}F NMR spectrum (470 MHz) in $CDCl_3$ with TTFB as standard

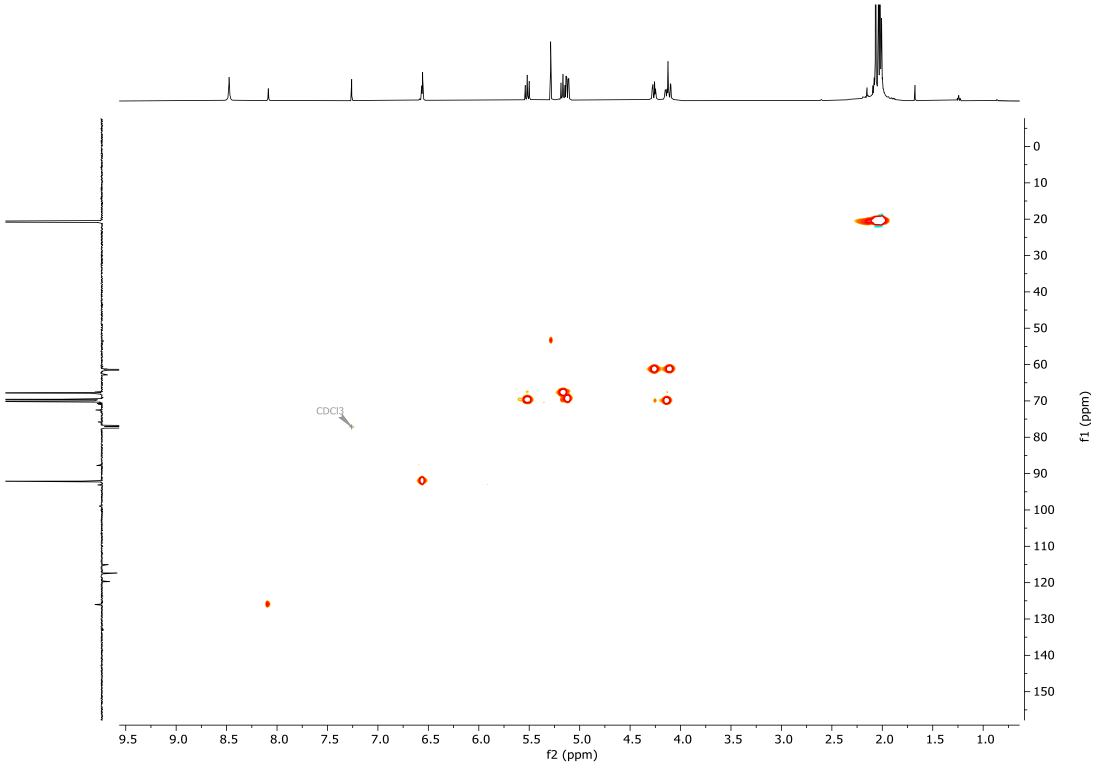

HSQC (500 MHz, 126 MHz) in $CDCl_3$

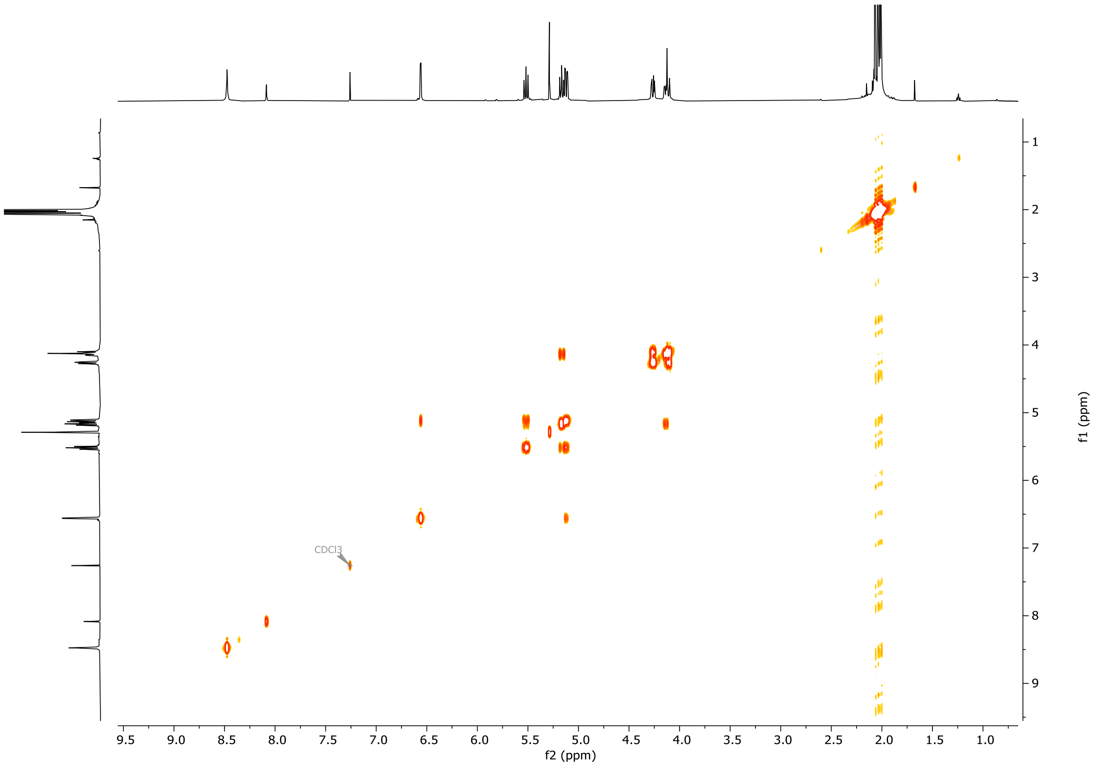

COSY (500 MHz) in $CDCl_3$

O-(2,3,4,6-Tetra-*O*-acetyl-α-D-glucopyranosyl) bromodifluoroacetimidate **6-α**

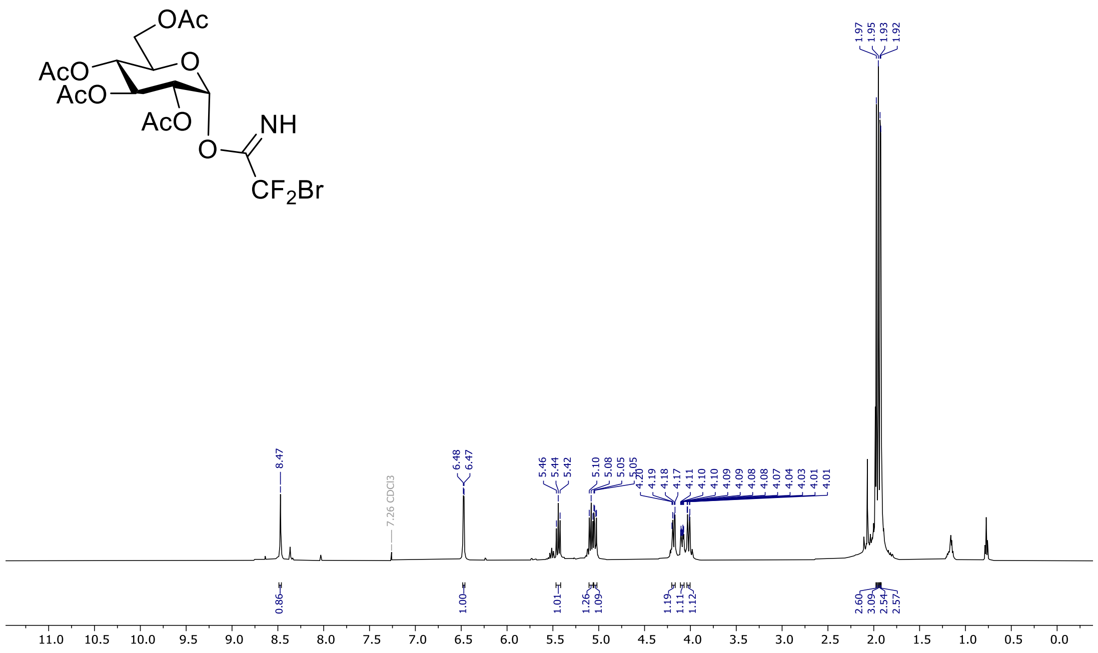

^{1}H NMR (500 MHz) in $CDCl_3$

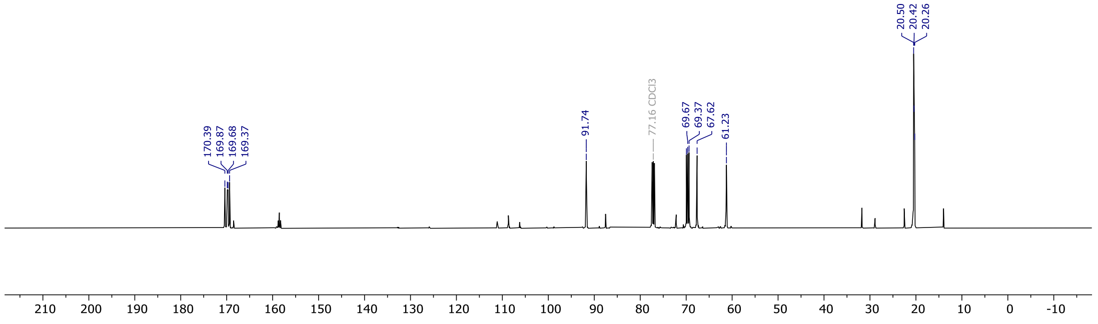

^{13}C NMR (126 MHz) in $CDCl_3$

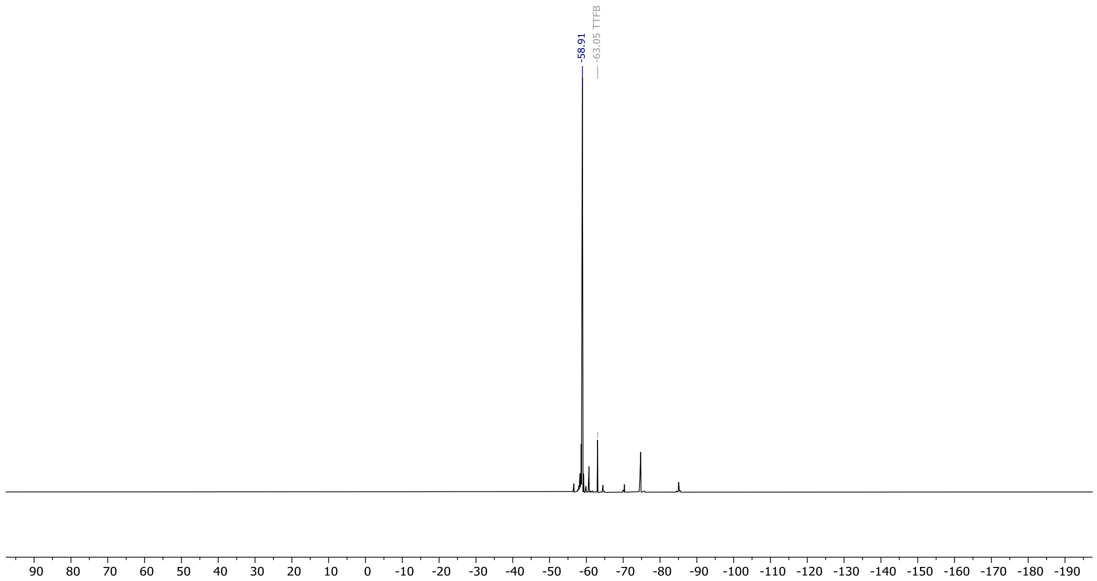

^{19}F NMR (470 MHz) in $CDCl_3$ with TTFB as reference

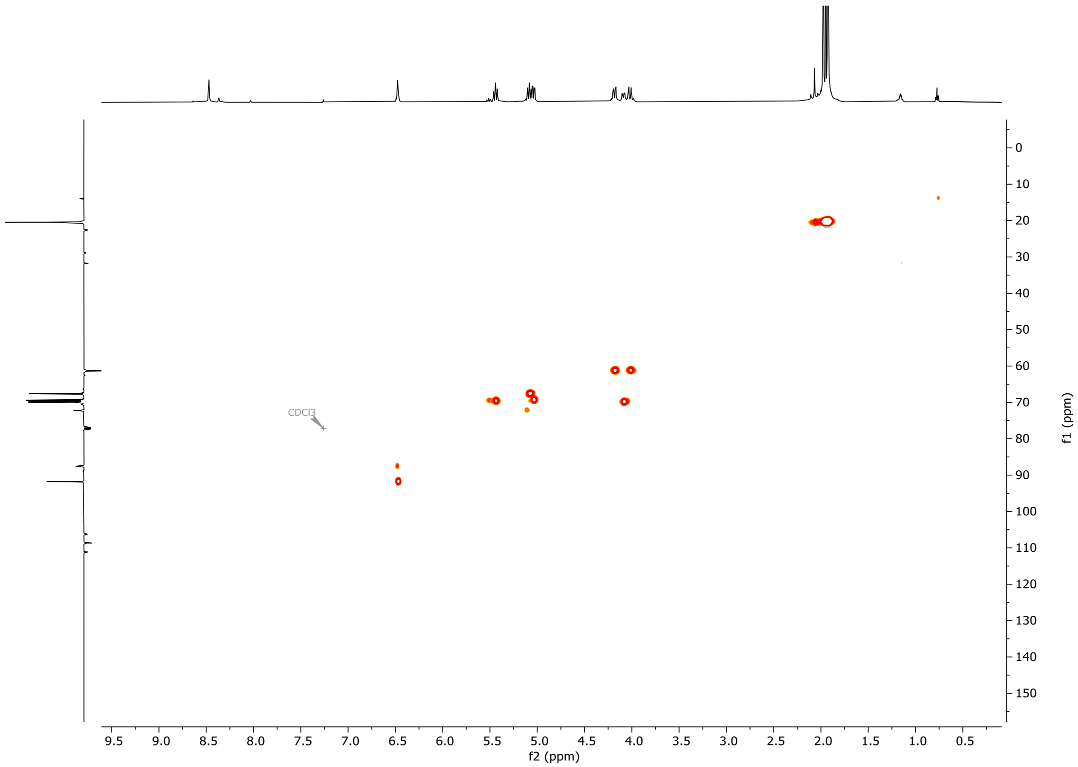

HSQC (500 MHz, 126 MHz) of compound **6** in $CDCl_3$

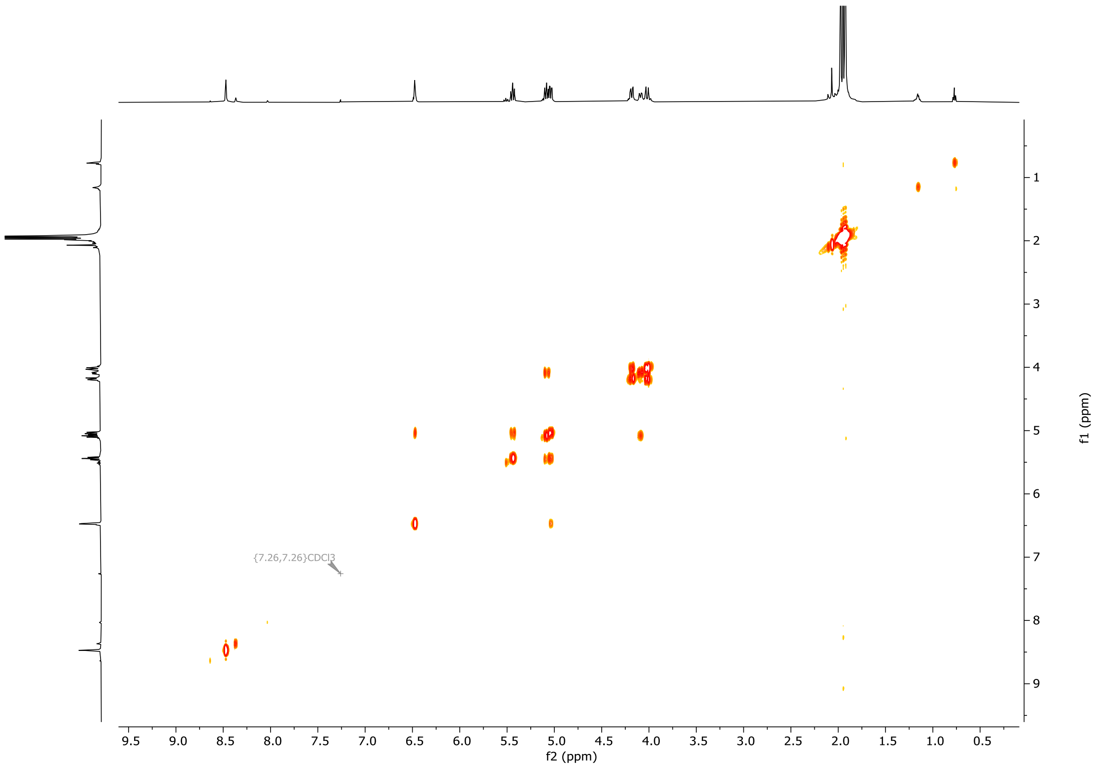

COSY (500 MHz) of compound **6** in $CDCl_3$

Compound **7**

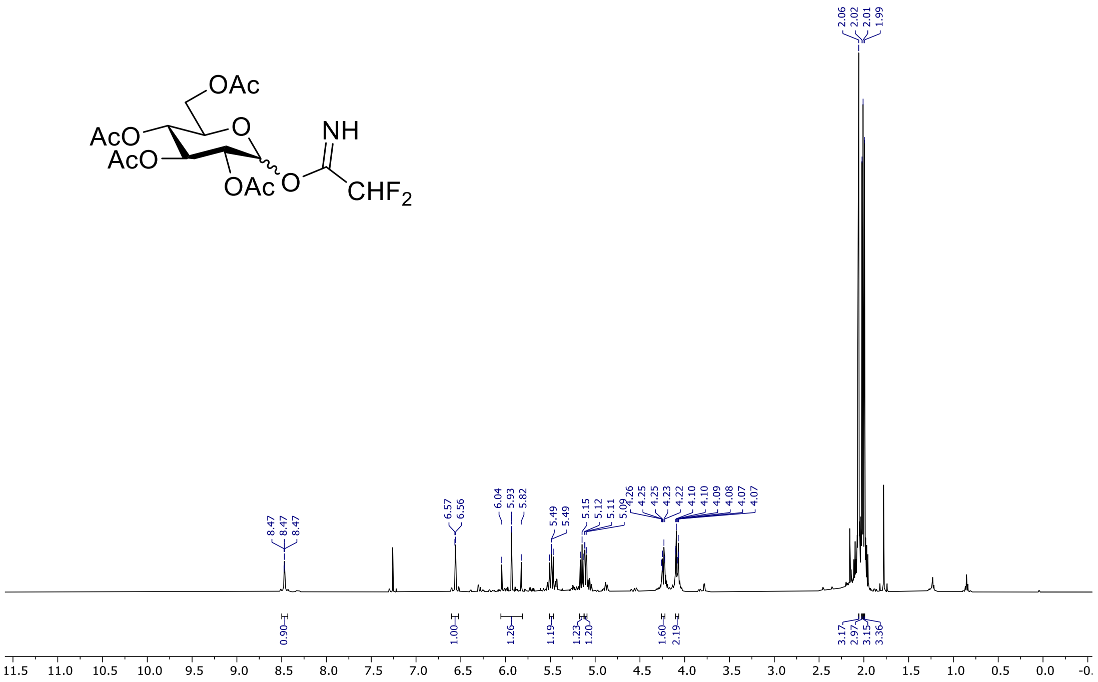

^{1}H NMR spectrum (500 MHz) in $CDCl_3$

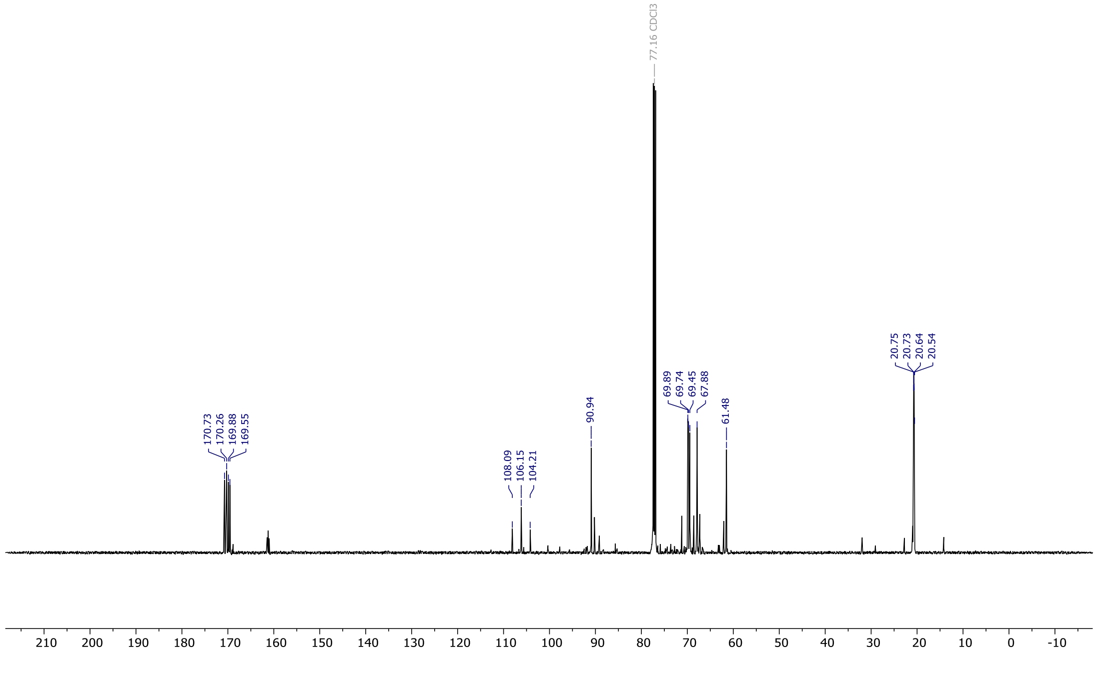

^{13}C NMR spectrum (126 MHz) in $CDCl_3$

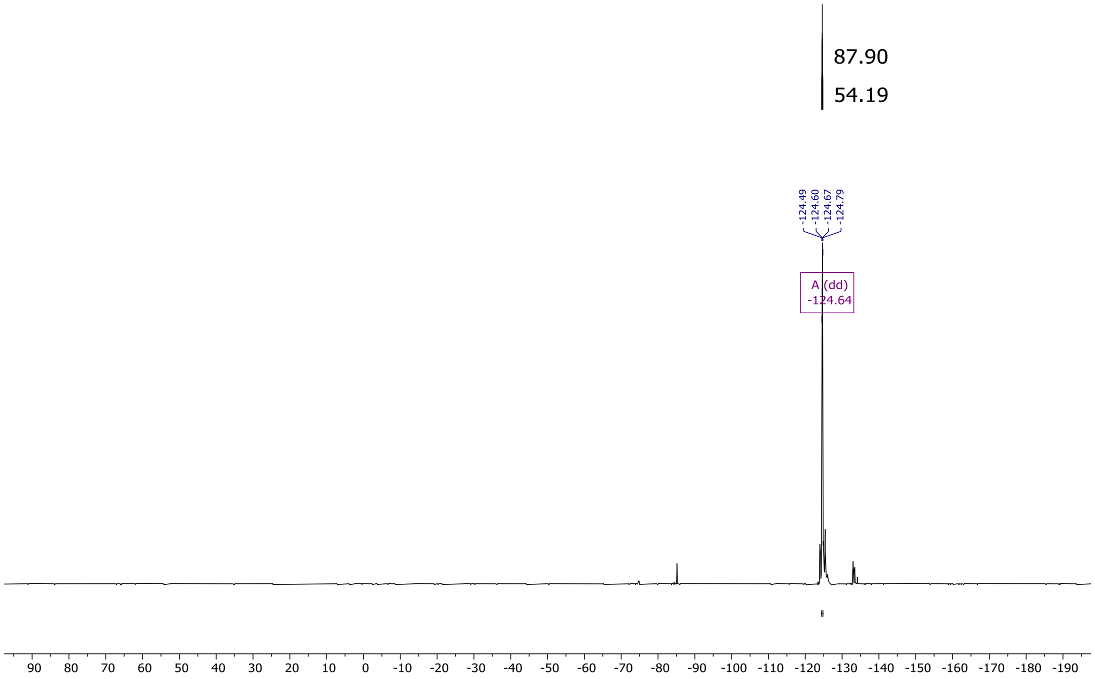

Unreferenced ^{19}F NMR spectrum (470 MHz) in $CDCl_3$

Naphthalen-2-ylmethyl 2,2,2-trifluoroacetimidate (**8**)

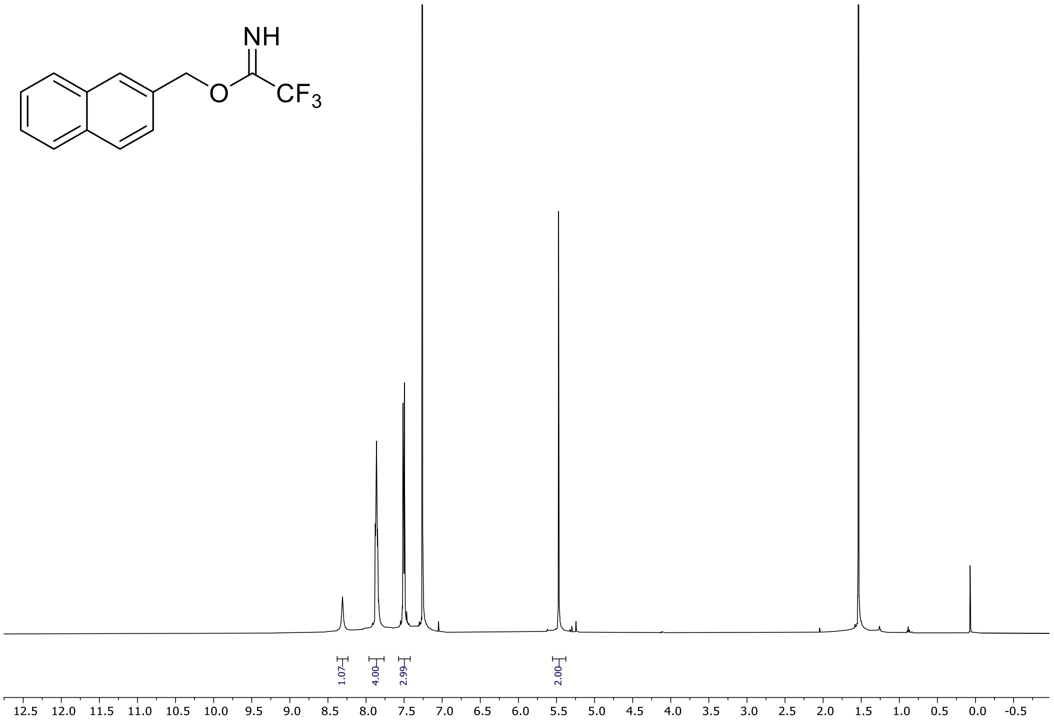

^{1}H NMR spectrum (500 MHz) in $CDCl_3$

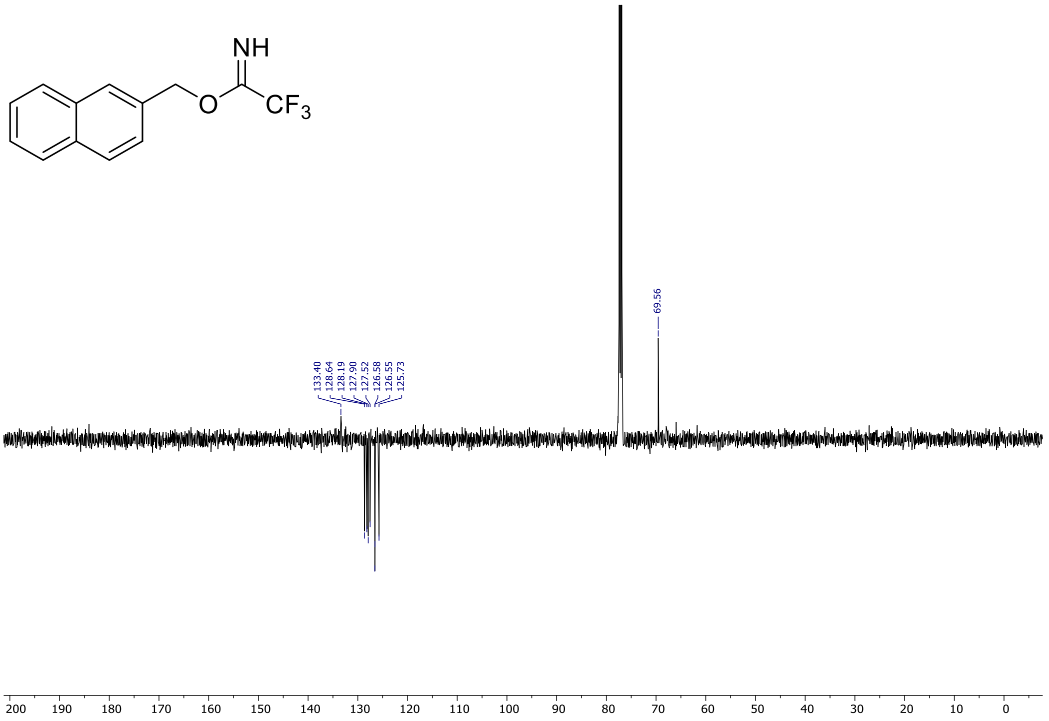

APT-^{13}C NMR spectrum (126 MHz) in $CDCl_3$

4-Methoxybenzyl 2,2,2-trifluoroacetimidate (**9**)

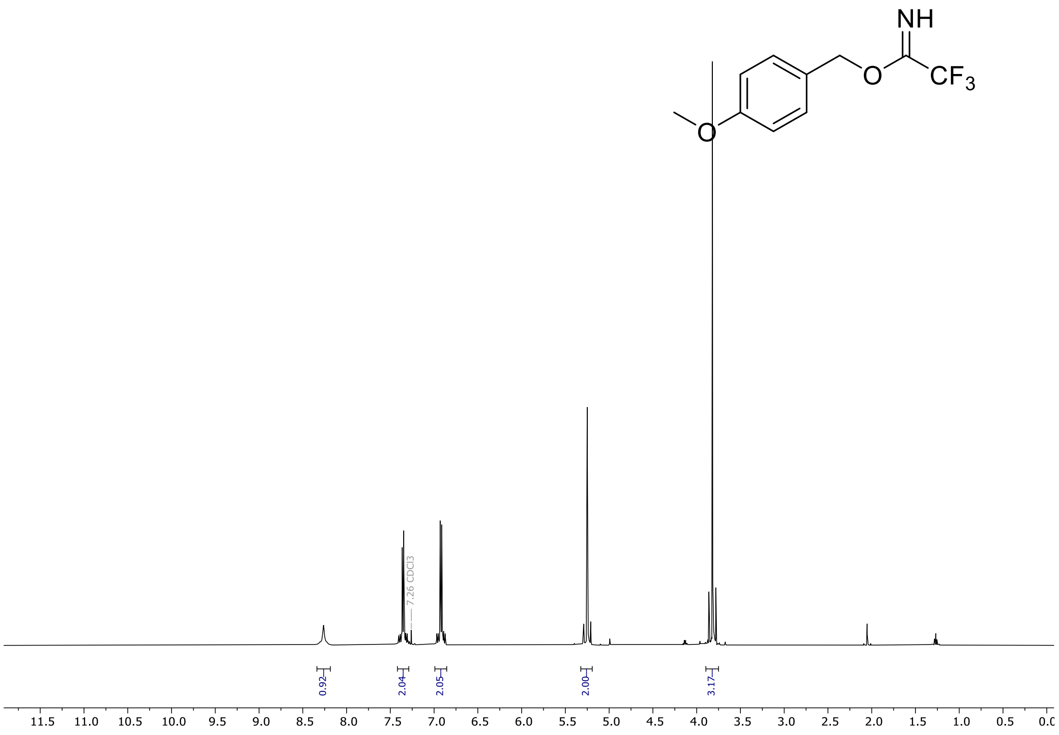

^1H NMR spectrum (500 MHz) in $CDCl_3$

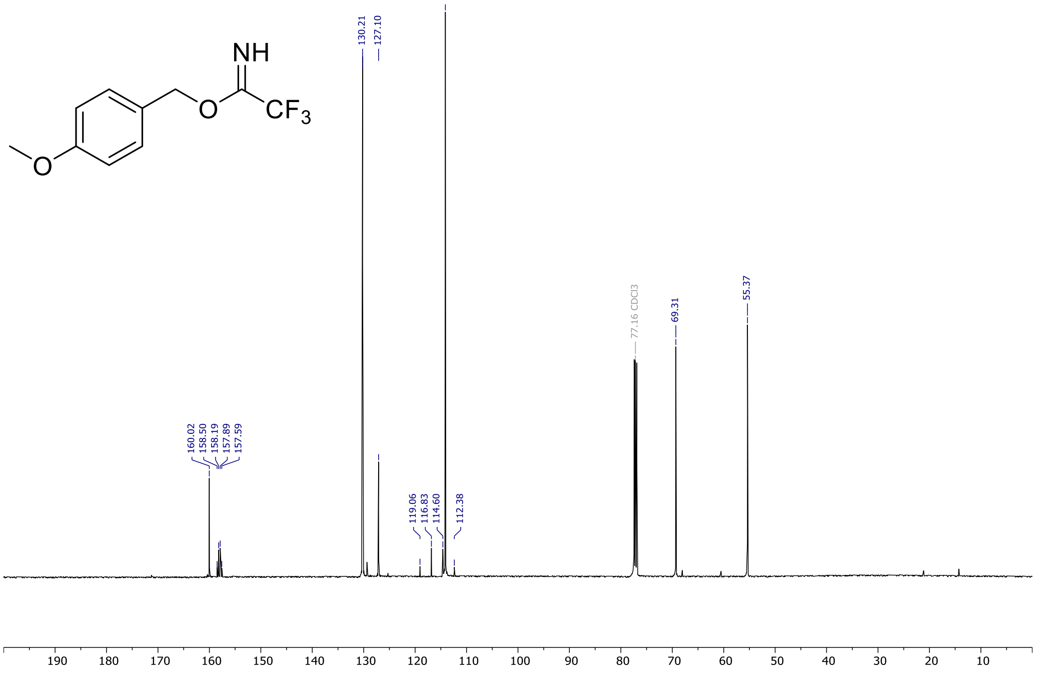

^{13}C NMR spectrum (126 MHz) in $CDCl_3$

4-Nitrobenzyl 2,2,2-trifluoroacetimidate (**10**)

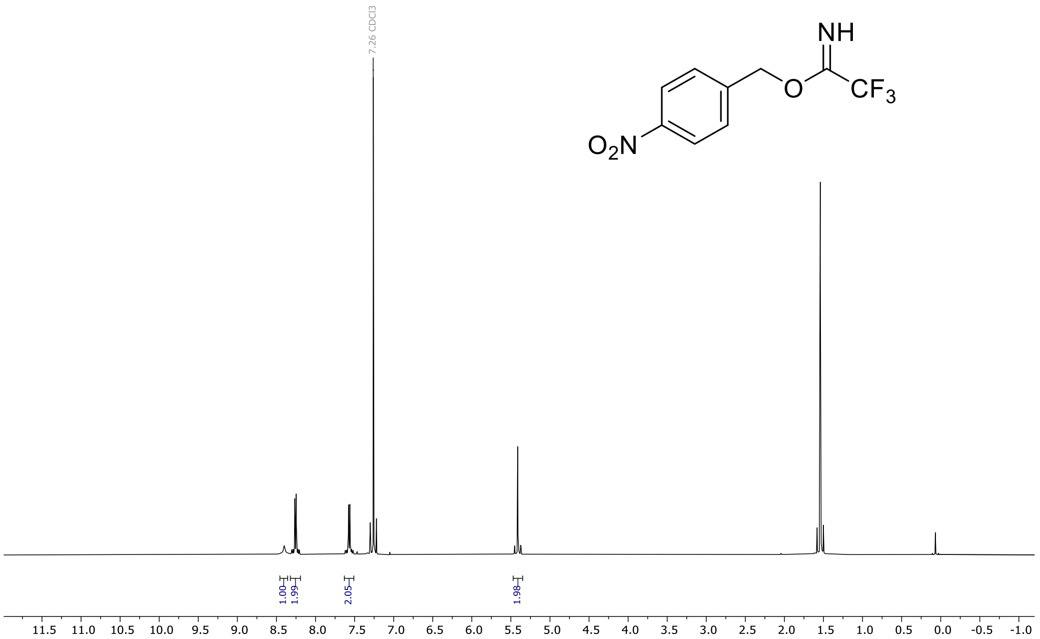

^{1}H NMR spectrum (500 MHz) in $CDCl_3$

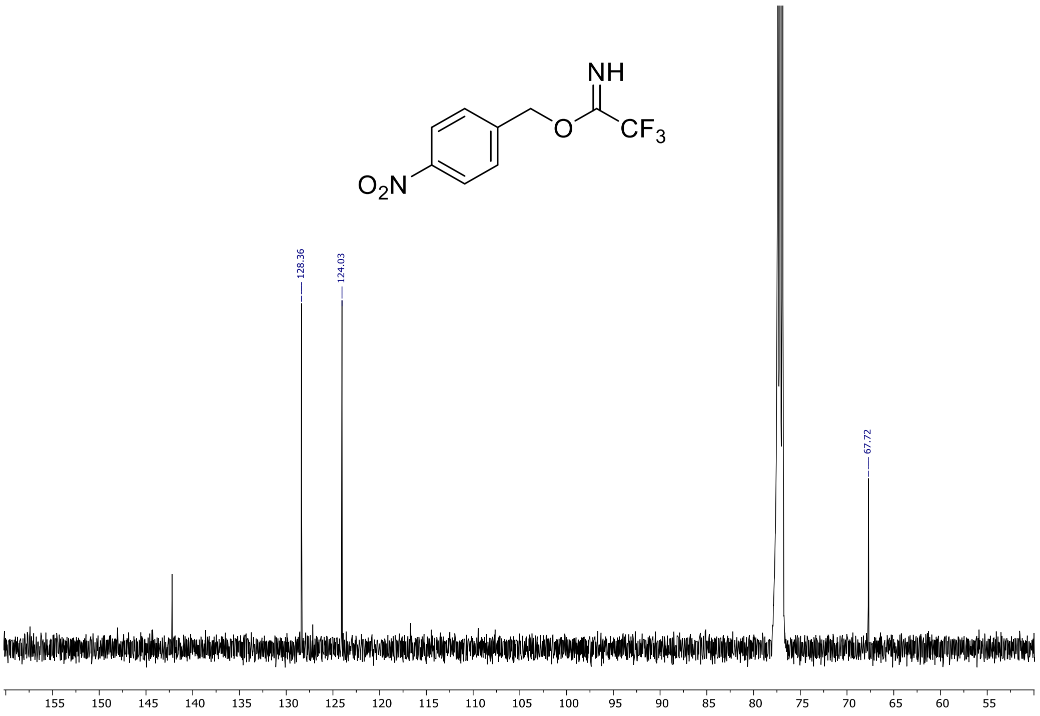

^{13}C NMR spectrum (126 MHz) in $CDCl_3$

4-Methoxybenzyl 2,2-difluoroacetimidate (**11**)

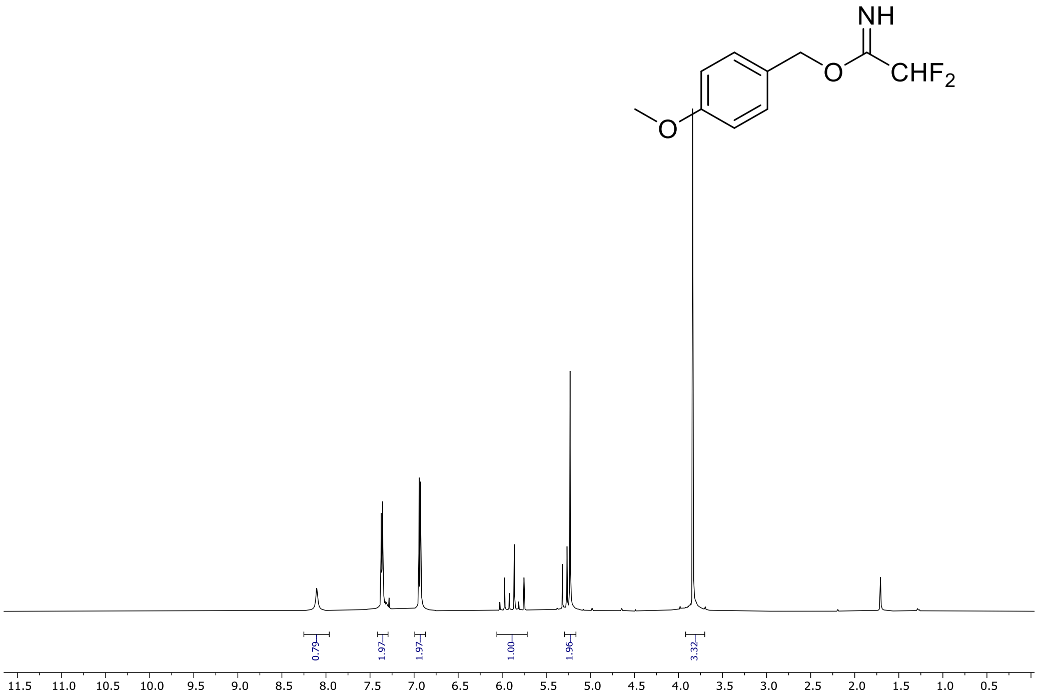

^{1}H NMR spectrum (500 MHz) in $CDCl_3$

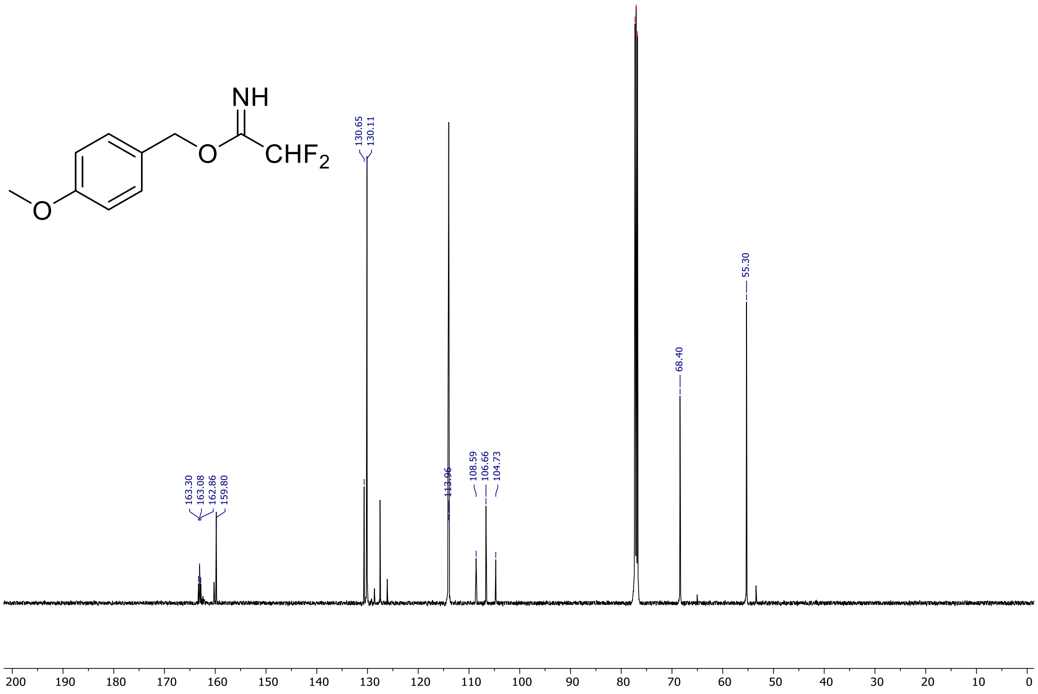

^{13}C NMR spectrum (126 MHz) in $CDCl_3$

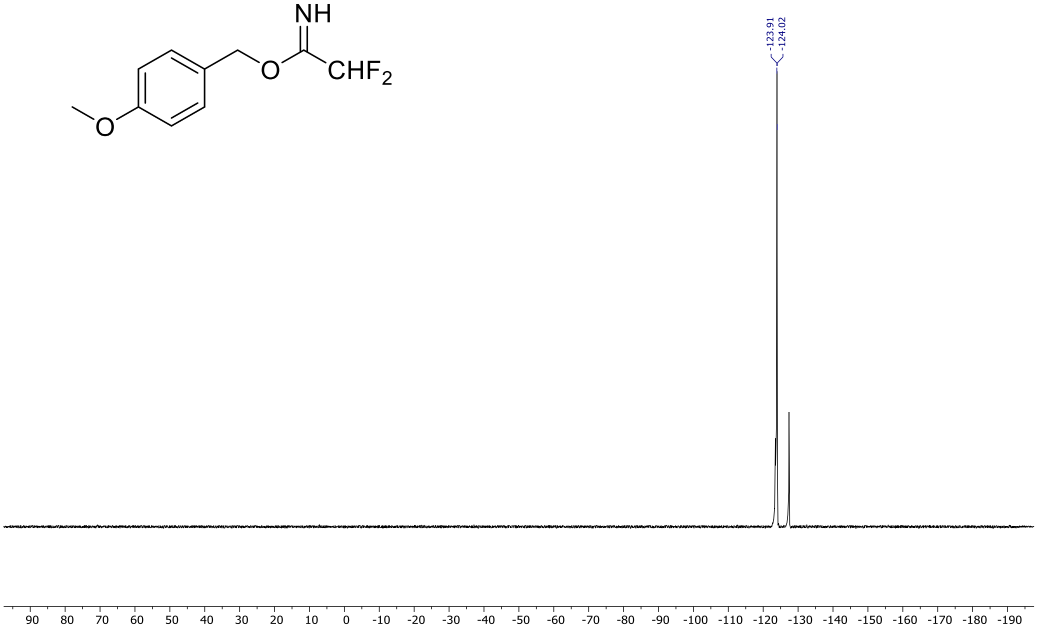

Unreferenced ^{19}F NMR spectrum (470 MHz) in $CDCl_3$
